# Druggable *Nucleolin* Identifies Breast Tumours Associated with Poor Prognosis That Exhibit Different Biological Processes

**DOI:** 10.3390/cancers10100390

**Published:** 2018-10-22

**Authors:** Flora Nguyen Van Long, Audrey Lardy-Cleaud, Susan Bray, Sylvie Chabaud, Thierry Dubois, Alexandra Diot, Lee B. Jordan, Alastair M. Thompson, Jean-Christophe Bourdon, David Perol, Philippe Bouvet, Jean-Jacques Diaz, Virginie Marcel

**Affiliations:** 1 Univ Lyon, Université Claude Bernard Lyon 1, INSERM 1052, CNRS 5286, Centre Léon Bérard, Centre de Recherche en Cancérologie de Lyon, CEDEX 08, 69373 Lyon, France; floranvl@gmail.com (F.N.V.L.); philippe.bouvet@ens-lyon.fr (P.B.); 2 Department of Clinical Research, Léon Bérard Cancer Centre, 28 rue Laennec, 69008 Lyon, France; audrey.lardy-cleaud@lyon.unicancer.fr (A.L.-C.); sylvie.chabaud@lyon.unicancer.fr (S.C.); david.perol@lyon.unicancer.fr (D.P.); 3 Tayside Tissue Bank, Ninewells Hospital and Medical School, NHS Tayside, Dundee DD1 9SY, Scotland, UK; s.e.bray@dundee.ac.uk; 4 Breast Cancer Biology Group, Translational Research Department, PSL Research University, Institut Curie, 26 rue d’Ulm, 75005 Paris, France; thierry.dubois@curie.fr; 5 Division of Cancer Research, University of Dundee, Ninewells Hospital and Medical School, Dundee DD1 9SY, Scotland, UK; A.Z.Diot@dundee.ac.uk (A.D.); alastair.thompson@bcm.edu (A.M.T.); j.bourdon@dundee.ac.uk (J.-C.B.); 6 Department of Pathology, University of Dundee, Ninewells Hospital and Medical School, Dundee DD1 9SY, UK; l.jordan@dundee.ac.uk; 7 Olga Keith Wiess Chair of Surgery, Dan L. Duncan Breast Center, Division of Surgical Oncology, Baylor College of Medicine, Houston, TX 77030, USA; 8 Ecole Normale Supérieure de Lyon, Université de Lyon, CEDEX 07, 69342 Lyon, France

**Keywords:** nucleolin, breast cancer, prognostic marker, triple-negative breast cancer

## Abstract

*Background:* Nucleolin (NCL) is a multifunctional protein with oncogenic properties. Anti-NCL drugs show strong cytotoxic effects, including in triple-negative breast cancer (TNBC) models, and are currently being evaluated in phase II clinical trials. However, few studies have investigated the clinical value of *NCL* and whether *NCL* stratified cancer patients. Here, we have investigated for the first time the association of *NCL* with clinical characteristics in breast cancers independently of the different subtypes. *Methods:* Using two independent series (*n* = 216; *n* = 661), we evaluated the prognostic value of *NCL* in non-metastatic breast cancers using univariate and/or multivariate Cox-regression analyses. *Results:* We reported that *NCL* mRNA expression levels are markers of poor survivals independently of tumour size and lymph node invasion status (*n* = 216). In addition, an association of *NCL* expression levels with poor survival was observed in TNBC (*n* = 40, overall survival (OS) *p* = 0.0287, disease-free survival (DFS) *p* = 0.0194). Transcriptomic analyses issued from The Cancer Genome Atlas (TCGA) database (*n* = 661) revealed that breast tumours expressing either low or high *NCL* mRNA expression levels exhibit different gene expression profiles. These data suggest that tumours expressing high *NCL* mRNA levels are different from those expressing low *NCL* mRNA levels. *Conclusions: NCL* is an independent marker of prognosis in breast cancers. We anticipated that anti-NCL is a promising therapeutic strategy that could rapidly be evaluated in high *NCL*-expressing tumours to improve breast cancer management.

## 1. Introduction

The expression level of hormonal and growth receptors remains one of the strongest prognostic biomarkers driving management of breast cancer. The identification of breast cancer subtypes, which present distinct prognosis associated with particular expression patterns of receptors, has indeed allowed the development of efficient anti-cancer therapies targeting the differentially expressed receptors [1]. However, triple-negative breast cancers that exhibit reduced expression of *Human Epidermal Growth Factor Receptor-2* (*HER2*) and hormonal oestrogen receptor (ER) and progesterone receptor (PR), are still an on-going challenge since the standard chemotherapy-based treatment has shown only limited objective response and elevated relapse events. Novel stratification’s markers and druggable targets are urgently needed for such breast cancer patients.

One opportunity could arise from nucleolin (NCL), a multifunctional protein. *NCL* is involved in several molecular processes, including regulation of ribosome biogenesis, transcription by RNA polymerase II, DNA repair or centrosome formation [2]. *NCL* is hence involved in numerous biological functions controlling cellular homeostasis through modulation of cell proliferation, apoptosis, and cell survival. Recent studies also demonstrated the role of *NCL* in angiogenesis, epithelial-to-mesenchymal transition, and stemness [3,4]. In agreement with *NCL* properties, several in vitro and in vivo studies had reported an oncogenic effect of *NCL* that appears to be multifactorial, reflecting its multiple functions [4].

Several drugs targeting NCL have been developed that specifically induced cell death of tumour cells [2,5,6]. Among them, two anti-NCL have been evaluated in clinical trials. Since the toxico-kinetic evaluation of the antagonistic peptide N6L showed accurate results on solid tumours during a phase I/IIa clinical trial (NCT01711398), a phase II is currently under preparation. In contrast, the NCL-targeted DNA aptamer AS1411 has shown only limited efficacy in a phase II clinical trial on unselected patients with advanced stage of renal cell carcinoma: only one patient out of the 35 enrolled ones showed dramatic reduction of tumour burden [7]. Interestingly, most of the anti-NCL therapies have been evaluated in vitro and in vivo using triple-negative breast cancer models and showed strong cytotoxic effects, making *NCL* a powerful candidate for future evaluation of anti-NCL in breast cancer [6]. Indeed, the antibody 4LB5 inhibiting NCL activity decreased MDA-MB-231 cell viability, clonogenicity, and tumour growth in xenografts while inducing apoptosis [8]. In addition, the peptides F3 and HB19 have been shown to significantly reduce tumour growth using MDA-MB-435 and MDA-MB-231 xenografts models, respectively [9,10]. Anti-NCL therapies could thus represent a novel opportunity for breast cancer, and in particular for triple-negative breast cancer patients.

At present, the role of *NCL* in cancer is supported only by few studies using human samples that showed the association of *NCL* expression with poor overall survival both in solid and liquid cancers [6,11,12,13]. In breast cancer, only two studies investigated *NCL* expression. It has been reported that *NCL* is often overexpressed in breast tumours compared to normal tissues and that *NCL* overexpression is associated with poor overall survival in HER2-amplified breast cancer [14,15]. Additional studies are required to evaluate more extensively the clinical value of *NCL* expression in order to understand the role of *NCL* in breast cancer.

## 2. Results

### 2.1. Association of NCL mRNA Levels with Overall and Disease-Free Survival in Breast Cancer

To evaluate the prognostic value of *NCL* in non-metastatic breast cancer, we quantified *NCL* mRNA expression levels by RT-qPCR in the Dundee series of 216 primary breast tumours (Table 1, All samples). Since we decided to perform survival association’s study without initial hypothesis regarding alteration of *NCL* in breast tumours, we performed preliminary study to determine a cut-off based on the distribution of *NCL* expression. The quartile distribution was first analysed (Figure S1A). We observed three groups of quartiles (Q2, Q3/Q4, and Q1). We thus used tercile distribution and confirmed a *NCL*-based stratification in three groups (Figure S1B). Finally, since we would like to identify only the true “low” and the true “high”, we thus divided patients in three groups bearing tumours expressing either low (0–20%), intermediate (20–80%), or high *NCL* levels (80–100%).

**Figure 1:**
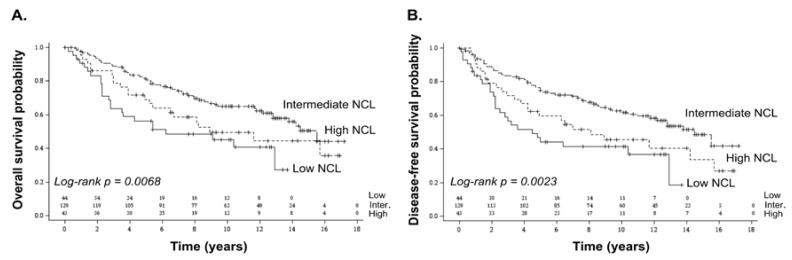
Association of *NCL* expression levels with poor prognosis in breast cancer using Dundee series. A significant association was observed between *NCL* expression levels and overall (**A**) as well as disease-free survivals (**B**). Association between *NCL* expression levels and survival was analysed using Kaplan–Meier representation. Number of subjects at risk is indicated on the graph for the three *NCL* groups (low; intermediate; high). Log-rank *p*-value ≤ 0.05 was used to determine significant association.

Using Kaplan–Meier survival curves and Log-rank tests, we observed that the expression levels of *NCL* were associated to overall and disease-free survivals (Log-rank: overall survival (OS) *p* = 0.0068; disease-free survival (DFS) *p* = 0.0023) (Figure 1A,B). Patients with tumours expressing high *NCL* levels had poorer overall and disease-free survival than patients with tumours expressing intermediate *NCL* levels. Unexpectedly, we also observed that patients with tumours expressing low *NCL* levels had poorer overall and disease-free survivals than patients with tumours expressing intermediate *NCL* levels. Similar significant associations were observed with both overall and disease-free survivals using univariate Cox regression models (*p* = 0.0083 and *p* = 0.0030, respectively) (Table S3). Importantly, the low *NCL*-expressing group was associated with a higher hazard ratio compared to the intermediate *NCL*-expressing group (Low: OS hazard ratio (HR) = 2.13, 95% confidence interval (95% CI) = (1.30–3.48); DFS HR = 2.20, 95% CI = (1.37–3.52)). A same tendency even if not significant is observed for the high-versus intermediate NCL-expressing group (High: OS HR = 1.49, 95% CI 95% = (0.90–2.46); DFS HR = 1.60, 95% CI = (1.00–2.58)) (Table S3). However, it has to be noted that these observations relied on a limited number of samples.

When compared to 11 healthy mammary tissues, *NCL* mRNA expression levels of the healthy donors were not significantly different from the low *NCL*-expressing group, while they were significantly lower than in the high *NCL*-expressing groups (Figure S2). It suggests that high *NCL* expression levels corresponded to *NCL* overexpression compared to normal tissues (Figure S2). A second independent series corresponding to a TCGA dataset of 661 primary breast tumours was used (Table S1). Since the TCGA dataset corresponds to RNA-seq data and Dundee dataset to RT-qPCR data, we could not compare their expression values nor use an absolute cut-off. Thus, cut-off defined using Dundee series was used for TCGA series. As for Dundee series, a similar tendency was observed, low and high *NCL* expression levels being associated with poor overall and disease-free survival (OS *p* = 0.1084; DFS *p* = 0.2658) (Figure S3). The lack of significance could arise from the short follow-up median of TCGA series (2.81 years; minimum (min): 0; maximum (max): 23.6) compared to the one of Dundee series (12.4 years; min: 0; max: 17.2) as well as from the difference in clinical characteristics, such as the variation of tumours proportion in intrinsic breast cancer subtypes (Table 1 and Table S1).

To definitively assess whether *NCL* is a prognostic marker in non-metastatic breast cancer, we performed multivariate Cox regression models using Dundee series on *NCL* mRNA expression levels and robust clinical prognostic factors, including tumour size, lymph node invasion status and intrinsic breast cancer subtypes. While the “intrinsic breast cancer subtypes” parameter remained non-significant, the other parameters (*NCL*, size, and lymph node invasion) remained in the model. *NCL* levels therefore remained associated with both overall and disease-free survivals even after adjustment on tumour size and lymph node invasion (Table 2). Interestingly, in overall survival, NCL allowed for discriminating the most aggressive breast tumours exhibiting large size at diagnosis (Figure S4). Indeed, this analysis suggests that patients who carry small breast tumours (<30 mm) expressing high *NCL* levels had a poorer overall survival compared to patients who carry small breast tumours but expressing intermediate *NCL* levels for example. *NCL* thus allows for improving the current breast cancer patient stratification based on tumour size. These data indicated that *NCL* mRNA expression level is an independent marker of prognosis, even when compared to the most robust clinical prognostic factors (tumour size and lymph node invasion status).

### 2.2. Characterization of Breast Tumours Expressing Either Low or High NCL mRNA Levels

Distribution of clinical parameters between the three groups of *NCL* expression was then compared to determine whether the breast tumours expressing low or high *NCL* levels, both associated with the poorest survivals, shared common traits (Table 1). While mean age, tumour size and invaded lymph node status at diagnosis showed no significant change in distribution among the three *NCL* groups, invasive grade and ER/PR status were significantly different. We noticed that tumours expressing low levels of *NCL* mRNA corresponded to 80% to grade 3 tumours, indicative of aggressive tumours with a high proliferation rate and poorly differentiated. In contrast, tumours expressing intermediate and high *NCL* levels corresponded to only 60% to grade 3 tumours. We also observed that the low *NCL*-expressing group mainly corresponded to patients carrying ER-negative tumours (about 57% compared to 26% for the intermediate- and high *NCL*-expressing groups). Finally, both low and high *NCL*-expressing groups mainly corresponded to patients with PR-negative tumours compared to the intermediate-*NCL*-expressing group (70.5% and 65.1% respectively compared to 46%). From the clinical point of view, low- and high *NCL*-expressing breast tumours appeared different.

To further compare tumours expressing low and high *NCL* levels, we analysed gene expression profiles in these different tumours. Although survival association with *NCL* expression was not significant in TCGA series but nevertheless showed the same tendency than Dundee series, we used TCGA series for such analysis since the whole transcriptome datasets are available. Clustering approaches revealed that four out of 10 gene-based clusters exhibited different expression profiles between tumours expressing low or high *NCL* levels (Figure S5). Among them, one cluster was enriched in transcription-related genes, whose expression was higher in tumours expressing high *NCL* levels compared to the ones expressing low *NCL* levels. This was expected knowing the role of NCL in regulating transcription and proliferation (Cluster 8, Figures S5 and S6, Table S4). Moreover, low *NCL* tumours expressed higher levels of genes involved in immunity and cell adhesion compared to high *NCL* tumours (Clusters 6 and 7, Figures S5 and S6, Table S4). Overall, these clinical and biological data suggested that tumours with low and high *NCL* expression levels corresponded to distinct types of tumours.

### 2.3. Prognostic Value of NCL in Breast Cancer Subtypes

**Figure 2:**
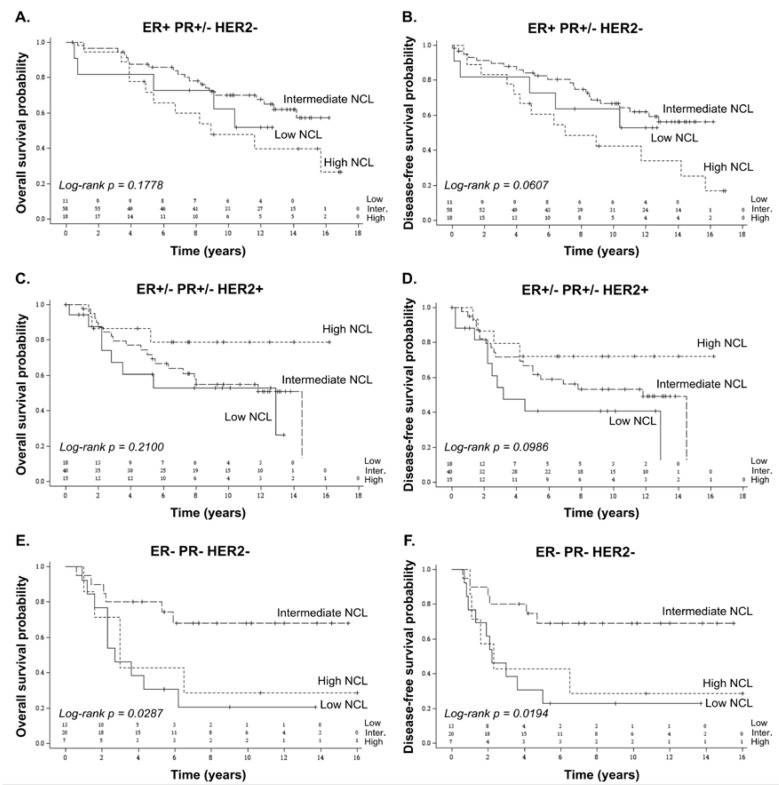
Association of *NCL* expression levels with poor prognosis in breast cancer subtypes using Dundee series. Overall (**A**,**C**,**E**) and disease-free survivals (**B**,**D**,**F**) in relation to *NCL* expression levels were analysed in tumours exhibiting different hormonal status: ER+ PR+/− HER2− (equivalent to the luminal subtype, **A**,**B**); ER+/− PR+/− HER2+ (equivalent to the HER2 subtype, **C**,**D**); and ER− PR− HER2− (equivalent to the triple-negative subtype, **E**,**F**). While *NCL* expression levels were not associated with survivals in patients carrying ER+ PR+/− HER2− and ER+/− PR+/− HER2+ tumours, associations were observed between *NCL* expression levels and both overall and disease-free survivals in patients bearing ER− PR− HER2− tumours. The association between *NCL* expression levels and survival was analysed using Kaplan–Meier representation. Number of subjects at risk is indicated on the graph for the three *NCL* groups (low; intermediate; high). Log-rank *p*-value ≤ 0.05 was used to determine significant association.

Since *NCL* mRNA expression level was associated with hormonal status, we investigated whether *NCL* prognostic value was dependent upon breast cancer subtype. Using Dundee series, Kaplan–Meier curves were plotted for the three main intrinsic breast cancer subtypes (ER+ PR+/− HER2− corresponding to the luminal subtype; ER+/− PR+/− HER2+ to a HER2-amplified subtype; and ER− PR− HER2− to a triple-negative subtype). The affiliation of each tumour to one of the three *NCL*-expressing groups (low, intermediate, high) was not re-evaluated for each subtype but conserved regarding the entire population. No significant association were observed between *NCL* expression levels and both overall and disease-free survivals in the ER− PR+/− HER2− and ER+/− PR+/− HER2+ breast cancer subtypes (Figure 2A–D). In contrast, a significant association, however based on a small number of TNBC patients (*n* = 40), was observed in ER− PR− HER2− breast cancer subtype (Figure 2E,F). Patients carrying ER− PR− HER2− breast tumours expressing high *NCL* levels had poorer overall and disease-free survivals compared to the ones carrying ER− PR− HER2− breast tumours expressing intermediate *NCL* levels. A similar tendency was observed for tumours expressing low *NCL* mRNA levels. Altogether, these data suggest that *NCL* could be a prognostic marker in triple-negative breast cancers.

## 3. Discussion

Although most of studies dedicated to determine the clinical value of *NCL* have been performed in neuroepithelial tumours, pancreatic ductal adenocarcinoma (PDAC), hepatocellular carcinoma (HCC), and leukaemia, few have been performed in breast cancer. Here, we show that *NCL* expression level is an independent marker of prognosis in breast cancer. Patients bearing tumours expressing high *NCL* mRNA levels, but also unexpectedly the ones expressing low *NCL* mRNA levels, had poorer overall and disease-free survivals than other breast cancer patients even when adjusted to two robust clinical factors, tumour size and lymph node invasion status. Moreover, it appears that tumours expressing low or high *NCL* levels corresponded to distinct tumours both from the clinical and biological point of views.

Up to now and as far as we know, only one study reported an association between *NCL* expression and overall survival in breast cancer. Using the TCGA dataset, Wolfson and colleagues showed that patients carrying HER2-amplified tumours with high *NCL* expression levels had shorter overall and disease-free survivals than the ones carrying HER2-amplified tumours with low *NCL* expression levels [14]. In our hand, no association was observed in HER2-amplified breast cancers in Dundee series. This discrepancy could first be explained by difference in clinical characteristics between Dundee and TCGA series, in particular regarding repartition of intrinsic breast cancer subtypes (ER+/− PR− HER2+: Dundee 36.5% vs. TCGA 21.8%) (Table 1 and Table S1), and choice of cut-off. Second, HER2 status in TCGA dataset was lacking or uncertain for several patients (about 226, Table S1). It might thus induce a bias regarding this specific cancer subtype in TCGA series. This is why we did not use TCGA series to determine whether *NCL* prognostic value was dependent upon breast cancer subtype.

A second study reported that *NCL* expression level was over-expressed in mammary tumours compared to normal tissues using both immunohistochemistry (IHC) in a panel of eight normal/tumour pairs and NanoString-quantified mRNA levels in 57 normal vs. 183 triple-negative breast cancer [15]. A recent review reported similar observations on small series [6]. These data are concordant with our observation that tumours of the high *NCL*-expressing group expressed more *NCL* mRNA levels than healthy mammary tissues. Interestingly, Pichiorri and colleagues also observed in a panel of 70 triple-negative breast cancers using IHC that, in addition to the 30% of tumours showing high NCL staining, NCL was not detected in about 20% of tumours [15]. Although we did not know why NCL was not detected in this study, this observation nevertheless supported the existence of tumours with low expression of *NCL*. We observed that low *NCL* breast tumours express similar mRNA levels than normal mastectomy tissues issued from a small cohort (*n* = 11). From this point of view, *NCL* exhibits the same characteristics as ER, PR, and HER2 biomarkers, for which negative scoring corresponds to expression similar to the that of normal tissue even though they are essential for clinical stratification of breast cancer. Pichiorri and colleagues did not analyse the survival of patients carrying mammary tumours expressing these different NCL protein expression levels, thus preventing comparison with our conclusion on survival association. Due to the limit of our data that only focused on *NCL* mRNA level but also on the limited numbers of breast tumour samples, further clinical studies will be required to analyse correlation of *NCL* mRNA and protein expression levels in breast tumours. In particular, additional studies will be required to demonstrate the existence of tumours lacking *NCL* expression and the association between low *NCL* mRNA levels and poor survival.

As observed in our data, it has been reported in a series of 69 stage II PDACs that low level of nucleoli *NCL* expression was a marker of poor prognosis independently of age at diagnosis, tumour size, differentiation, and lymph node invasion status [16]. These data support the association of low *NCL* expression levels with poor survival after adjustment with robust prognostic factors, at least in some cancer types. Indeed, in other cancer types, such as HCC, neuroepithelial cancers, lung cancers, and acute myeloid leukaemia, high *NCL* expression levels have been associated with poor survival [11,12,17,18]. Overall, it suggests that low and high *NCL* expression levels can be marker of poor prognosis depending on the biological context. Interestingly, the series used in our study are enriched in early breast cancer. Indeed, more than 85% of the breast cancer patients present a tumour size that is inferior to 50 mm size which is generally used to define early breast cancers. Altogether, it suggests that low and high *NCL* expression levels could be a prognostic marker of early breast cancer able to identify at early stage, patients with poor prognosis.

In vitro and in vivo studies revealed that oncogenic effects of NCL can have multimodal origins reflecting the multifunctional properties of NCL in the different subcellular compartments [4]. Following this line, it has been reported that decrease of *NCL* mRNA levels mainly reduced expression of surface and cytoplasmic NCL protein without affecting the one of nuclear NCL [5], suggesting that depending on *NCL* expression levels distinct functions of NCL could be involved. Our data indicated that mammary tumours expressing low or high *NCL* levels had different clinical characteristics (i.e., tumour grade and ER/PR hormonal status), supporting association with distinct *NCL*-dependent functions. Transcriptomic analysis indeed revealed that low *NCL* tumours expressed high levels of genes involved in immunity and cell adhesion compared to high *NCL* tumours. Interestingly, we recently reported that *NCL* silencing was associated with an up-regulation of genes involved in inflammation response in HeLa cells and with increased number of centrosome-like structures in U2OS cells [19,20].

Overall, our data open up interesting opportunities in clinics. First, several promising anti-NCL drugs have been developed to fight liquid and solid cancers that are currently evaluated in clinical trials phase II [2,5,6,7,8,9,10]. Compared to intermediate and low *NCL*-expressing tumours, tumours expressing high *NCL* are the most prone to respond to anti-NCL drugs. Identifying high *NCL*-expressing tumours could thus be viewed as an indispensable companion test for anti-NCL drugs, in particular in TNBC for which efficient therapy is still lacking. Based on the poor survival rate associated with low *NCL*-expressing tumours, one could be afraid that reducing NCL activity in tumours would have deleterious effects. However, our data suggest that tumours expressing low and high *NCL* levels exhibit not reverse but distinct clinical characteristics and gene expression patterns. Thus, we can expect that inhibition of NCL activity in high *NCL*-expressing tumours will not promote acquisition of a “low *NCL*-expressing tumour”-like phenotype but rather cell death, as reported in numerous studies. Second, the discovery that *NCL* is an independent marker of prognosis even after adjustment on the current gold-standard factors used in clinics for prognosis suggests that *NCL* could be an interesting biomarker for treatment decisions. Indeed, one of the remaining issues in breast cancer is to identify patients that exhibit poor survival even when presenting the “good” prognostic factors at diagnosis (i.e., small tumour size, no lymph node, or non-TNBC breast cancer subtypes). Such patients could benefit from treatment protocols usually reserved to more aggressive tumours. In this line, our multivariate analyses suggest that *NCL* could be used as a marker to improve treatment decision. For example, patients with small tumour size at diagnosis exhibit different survival rates depending on the *NCL* expression levels (Figure S4). Patients expressing low or high *NCL* levels showed a poorer survival rate than the ones expressing intermediate *NCL* levels, suggesting that the former could benefit from treatment protocol usually reserved for large tumours. In combination with the current gold-standard markers, the usage of *NCL* as biomarker could improve the current patient management. Moreover, since tumours expressing low *NCL* levels showed an increased expression in genes involved in inflammation and immunity, these tumours might benefit from the emerging immuno-therapies.

## 4. Materials and Methods

### 4.1. Human Breast Cancer and Healthy Donor Samples

A series of 216 total RNA issued from primary breast tumours were collected at diagnosis from untreated female patients without metastasis and snap-frozen after macrodissection by a breast cancer pathologist (Tayside Tissue Bank (TTB), Dundee, Scotland, UK) [21]. Histological tumour grades and lymph node invasion were determined by breast cancer pathologists (Table 1). Hormonal status were determined by immunohistochemistry on sections of FFPE tumours [21]. Clinical data were collected and maintained by the TTB (last update 2016) that received ethical approval (REC Reference 07/S1402/90). A second series of 661 samples was extracted from the “Breast Invasive Carcinoma (TCGA, *Cell* 2015)” dataset of the public database cBioPortal on the basis of the following characteristics: female patients, primary breast cancer, no metastasis at diagnosis, no family history, no neoadjuvant treatment, and availability of RNA expression profile (Table S1) [22]. A third series of 11 healthy mammary tissues obtained from plastic surgery has been collected [23]. This series was approved by the Bioethic Law No. 2004-800, the Ethic Charter from the French National Institute of Cancer (INCa), and the ethics committee of Institut Curie.

### 4.2. RT-qPCR Analyses

RT was carried out using M-MLV Reverse Transcriptase enzyme (Invitrogen, Waltham, MA, USA). *NCL* expression levels were quantified by real-time qPCR using BioMark HD System (Fluidigm, South San Francisco, CA, USA) (Table S2) (See Supplementary Materials and Methods for more details). Relative fold-changes were calculated using the 2^−ΔΔCT^ method, using Human XpressRef Universal Total RNA (Qiagen, Hilden, Germany) as control sample and *GAPDH* as housekeeping gene (Figure S7). Expression levels were measured on two independent sets of triplicates.

### 4.3. Statistical Analyses

Cut-offs of *NCL* expression levels were determined using quartile/tercile-based overall survival. Descriptive statistics were used to summarize patients’ initial characteristics. Between-group comparisons were performed using a Chi-2’s or Fisher’s exact test for categorical data and *t*-test or nonparametric Kruskal–Wallis’ test for continuous data. Overall survival (OS) corresponded to timing from date of diagnosis to either death or last follow-up for censored patients. Disease-free survival (DFS) corresponded to timing from date of diagnosis to either relapse, death (if no relapse had been observed) or last follow-up for censored patients. Survival curves for OS and DFS with associated log-rank tests were generated using the Kaplan Meier method. Cox proportional hazards model was used to investigate confounding factors predicting for OS and DFS. In order to adjust for confounding factors a multivariate Cox models was performed. Variables sufficiently informed (less than 10% missing value) and significant at a 10% level in univariate analyses were included in a backward selection procedure to keep factors significant at a 5% level in the final multivariate model. All *p*-values corresponded to two-tailed *p*-values. A *p*-value ≤ 0.05 was considered statistically significant. Statistical analyses were performed using either SAS v9.4 (SAS Institute, Cary, NC, USA) or GraphPad Prism v7.0a softwares (GraphPad Software, Inc., La Jolla, CA, USA).

### 4.4. Transcriptomic Analyses

In addition to the clinical data and *NCL* expression extracted from the TCGA database, mRNA expression z-scores (RNA Seq V2 RSEM) were extracted from database cBioPortal for Cancer Genomics for the 661 identified breast tumours then divided in the three groups based on *NCL* expression (low, intermediate and high). Two-rounds of clustering analyses were performed using k-means clustering approach (See Supplementary Materials and Methods for additional details). A first clustering was performed independently in the three groups of *NCL* expression to identify tumours within the three *NCL*-based groups that exhibit similar expression profiles. A second clustering was performed on the three groups by conserving the previously identified tumour clustering to identify putative difference in gene expression between the three *NCL*-based breast tumours. Finally, gene ontology was performed on gene list issued from each cluster identified in this double k-mean clustering using DAVID tools (functional annotation clustering) [24].

## 5. Conclusions

Although breast cancer management has greatly improved in the last decade, a successful therapeutic strategy is still lacking for some breast cancer patients. In this study, we report that *NCL* is an independent prognostic marker in non-metastatic breast cancers. Our data bring the first clinical clue that *NCL* could be not only an useful biomarker to better stratify breast cancer patients when combined with the current gold-standard biomarkers, but also a promising target for *NCL*-overexpressing breast tumours to improve breast cancer management.

## Figures and Tables

**Table 1:** Population distribution of Dundee series regarding nucleolin (*NCL*) expression levels.

Characteristics	All Samples (*n* = 216)	Low *NCL* (*n* = 44)	Intermediate *NCL* (*n* = 129)	High *NCL* (*n* = 43)	*p*-Value
**Age**					
Median	59.0	58.5	59.0	60.0	0.557 ^#^
(min–max)	(28–90)	(28–78)	(31–90)	(35–88)	
**Invasive grade**					
No data	5	2	2	1	
Grade 1	14 (6.6%)	0 (0.0%)	8 (6.3%)	6 (14.3%)	0.013 ^$^
Grade 2	59 (28.0%)	7 (16.7%)	42 (33.1%)	10 (23.8%)	
Grade 3	138 (65.4%)	35 (83.3%)	77 (60.6%)	26 (61.9%)	
**Tumour size (mm)**					
*n*	204	42	120	42	
Median	28.0	28.5	28.0	26.0	0.641 ^#^
(min–max)	(5–110)	(11–80)	(7–110)	(5–110)	
**Invaded lymph node**					
No (*n* = 0)	98 (45.4%)	21 (47.7%)	58 (45.0%)	19 (44.2%)	0.936 ^†^
Yes (*n* ≥ 1)	118 (54.6%)	23 (52.3%)	71 (55.0%)	24 (55.8%)	
**ER status**					
No data	2	0	2	0	
Negative	70 (32.7%)	25 (56.8%)	34 (26.8%)	11 (25.6%)	<0.001 ^†^
Positive	144 (67.3%)	19 (43.2%)	93 (73.2%)	32 (74.4%)	
**PR status**					
No data	2	0	2	0	
Negative	117 (54.7%)	31 (70.5%)	58 (45.7%)	28 (65.1%)	0.005 ^†^
Positive	97 (45.3%)	13 (29.5%)	69 (54.3%)	15 (34.9%)	
**HER2 status**					
No data	16	2	11	3	
Negative	127 (63.5%)	24 (57.1%)	78 (66.1%)	25 (62.5%)	0.579 ^†^
Positive	73 (36.5%)	18 (42.9%)	40 (33.9%)	15 (37.5%)	
**Intrinsic breast cancer subtype**					
No data	16	2	11	3	
ER+ PR+/− HER2−	87 (43.5%)	11 (26.2%)	58 (49.2%)	18 (45.0%)	0.105 ^†^
ER+/− PR+/− HER2+	73 (36.5%)	18 (42.9%)	40 (33.9%)	15 (37.5%)	
ER− PR− HER2−	40 (20.0%)	13 (31.0%)	20 (16.9%)	7 (17.5%)	

^#^ Kruskal-Wallis test, ^$^ Fisher exact test, ^†^ Chi-2 test. PR: progesterone receptor; ER: oestrogen receptor.

**Table 2:** Multivariate Cox models in Dundee series.

Variable		Overall Survival			Disease-Free Survival		
		HR	95% CI	*p*-Value	HR	95% CI	*p*-Value
*NCL*	Intermediate	1.00			1.00		
	Low	2.31	(1.39–3.85)		2.32	(1.43–3.78)	
	High	1.55	(0.93–2.58)	0.0044	1.67	(1.03–2.71)	0.0019
Tumour size	<30 mm	1.00			1.00		
	≥30 mm	2.53	(1.66–3.85)	<0.0001	2.17	(1.46–3.23)	0.0001
Invaded lymph nodes	*n* = 0	1.00			1.00		
	*n* ≥ 1	1.60	(1.04–2.46)	0.0320	1.68	(1.12–2.53)	0.0130

HR: hazard ratio; 95% CI: 95% confidence interval.

## Supplementary Materials

The following are available online at http://www.mdpi.com/2072-6694/10/10/390/s1, Figure S1: Cut-off determination for *NCL* expression using the Dundee series, Figure S2: Comparison of *NCL* expression levels in tumours and healthy donors, Figure S3: Association of *NCL* expression levels with survivals of breast cancer patients using the TCGA series, Figure S4: Discrimination of the most aggressive breast tumours with large size at diagnosis using *NCL* expression, Figure S5: Comparison of gene expression profiles between the three *NCL* expressing groups of breast tumours in TCGA series, Figure S6: Gene ontology analysis on the clusters for which gene expression is different between low and high *NCL*-expressing breast tumours, Figure S7: Determination of the most stable housekeeping gene in our study to normalize RT-qPCR analyses, Table S1: Characteristics of patients issued from the TCGA dataset, Table S2: Nucelotide sequences of primers used in the study, Table S3: Univariate Cox regression models for survival in the Dundee series, Table S4: Full gene ontology analysis.
