# Supplementary material for: Druggable Nucleolin Identifies Breast Tumours Associated with Poor Prognosis That Exhibit Different Biological Processes
Source: Cancers (Basel). 2018 Oct 22;10(10):390. doi: 10.3390/cancers10100390 (PMC6210205; doi:10.3390/cancers10100390)
Supplement: Supplementary file 1 [file cancers-10-00390-s001.zip › cancers-343846 - supplementary - final.pdf]

Supplementary Materials: Druggable *Nucleolin* Identifies Breast Tumours Associated with Poor Prognosis that Exhibit Different Biological Processes

Flora Nguyen Van Long, Audrey Lardy-Cleaud, Susan Bray, Sylvie Chabaud, Thierry Dubois, Alexandra Diot, Alastair M. Thompson, Jean-Christophe Bourdon, David Perol, Philippe Bouvet, Jean-Jacques Diaz and Virginie Marcel

1. Supplementary Materials and Methods

1.1. Human Breast Cancer and Healthy Donor Samples

The first series (termed Dundee series) corresponds to a series of 216 primary breast tumours issued from the Tayside Tissue Bank (TTB, Dundee, Scotland, UK). Tumours were collected at diagnosis from untreated female patients without metastasis and snap-frozen after macrodissection by breast cancer pathologists [1]. At diagnosis, these patients showed no family history. Histological tumour grades and lymph node invasion were determined by breast cancer pathologists (Table 1). Grades were assigned from 1 to 3 based on their differentiation and proliferative status. Lymph node invasion was assigned as positive when at least one invaded lymph node was identified at diagnosis. Hormonal status (ER: oestrogen receptor; PR: progesterone receptor) and HER2 receptor were determined by immunohistochemistry on sections of FFPE tumour samples [1]. Hormonal status was used to identify the three intrinsic breast cancer subtypes: ER+ PR+/– HER2–, considered as equivalent to luminal subtype; ER+/– PR+/– HER2+, equivalent to HER2-amplified subtype; and ER– PR– HER2–, as triple-negative subtype (TNBC). Clinical data were collected and maintained by the TTB (last update 2016). The Dundee series exhibited expected clinical characteristics, in particular association of overall survival with tumour size, lymph node invasion and intrinsic breast cancer subtypes, as already described [1]. TTB has received ethical approval for samples and data collection (REC Reference 07/S1402/90). Total RNA were extracted from frozen tissues as already described by TTB services [1]. RNA quality was determined using the Bioanalyzer 2100™ (Agilent Technologies): samples for which the 28S/18S ratio was inferior to 1.2 were not included in this study as already reported [1].

The second series (termed TCGA series) corresponds to a series extracted from the "Breast Invasive Carcinoma (TCGA, *Cell* 2015)" dataset of the public database cBioPortal for Cancer Genomics [2]. From the 818 available samples in the dataset, we compiled a series of 661 tumours with the following characteristics: female patient, primary breast cancer, no metastasis at diagnosis, no family history, no neoadjuvant treatment and availability of RNA expression profiles (determined by RNA-seq analysis). However, among the 661 samples, some data were missing regarding hormonal status and relapse for example, explaining the difference of total sample number in Kaplan-Meier curves (Table S1 and Figure S3). For RNA expression, mRNA expression z-scores (RNA Seq V2 RSEM) was used as normalized quantification.

A series of 11 healthy mammary tissues obtained from plastic surgery, have been collected [3]. Total RNA were extracted from frozen tissues as previously described.

1.2. RT-qPCR Analyses

200ng of total RNA were retro-transcribed (RT) using M-MLV Reverse Transcriptase enzyme (Invitrogen, Waltham, MA, USA). *NCL* expression levels were quantified by real-time qPCR using BioMark HD System (Fluidigm, South San Francisco, CA, USA). A multiplex PCR was first performed on cDNA using genes of interest primers (Table S2) and Pre-Amp Master Mix (Fluidigm), followed by an Exonuclease I treatment (New England BioLabs, Ipswich, MA, USA) according to the Fluidigm instructions. After this first step of pre-amplification, cDNA were diluted at 1:5 in Tris-EDTA buffer 1× (Promega, Fitchburg, WI, USA). Gene expression was quantified using

Master Mix 2× EvaGreen (BioRad, Hercules, CA, USA), GE 48.48 Dynamic Array DNA Binding Dye Sample & Assay Loading Reagent kit (Fluidigm) and primers detailed in Table S2, as already described [4]. Relative fold-changes were calculated using the $2^{-\Delta\Delta CT}$ method by the Fluidigm Real-Time PCR analysis software (v3.1.3, Fluidigm), using Human XpressRef Universal Total RNA (Qiagen, Hilden, Germany) as control sample for inter-run normalization and *GAPDH* as housekeeping gene. RefFinder tool was used to identify the most stable gene among three potential housekeeping genes [5]. Each sample was subjected to two independent rounds of RT and each RT products were quantified in triplicate in two independent runs of qPCR. Statistical analyses were performed using mean values.

1.3. Statistical Analyses and Graphical Representations

Cut-offs of *NCL* expression levels were determined using population distribution-based overall survival in the Dundee series of 216 patients. To perform an analysis without hypothesis regarding the association of *NCL* expression with survival association and identify cut-off, the quartile distribution was first analysed. Since we observed 3 groups (Q2, Q3/Q4 and Q1), a tercile distribution was used. Furthermore, the tercile distribution allowed to gain into statistical power, thus allowing to reinforce our conclusion. Finally, since we would like to identify only the true "low" and the true "high", we used the population distribution based on 3 groups: tumours expressing low *NCL* expression levels that corresponds to [0–20%] of *NCL* expression levels; tumours expressing intermediate *NCL* expression levels that corresponds to]20–80%] of *NCL* expression levels; and tumours expressing high *NCL* expression levels that corresponds to]80–100%] of *NCL* expression levels. The same cut-off was applied on the TCGA series. Descriptive statistics were used to summarize patients' initial characteristics. Between-group comparisons were performed using a Chi-2's or Fisher's exact test for categorical data and *t* test or non-parametric Kruskal-Wallis' test for continuous data. Overall survival (OS) corresponded to the timing from date of diagnosis to either death or last follow-up for censored patients. Disease-free survival (DFS) corresponded to the timing from date of diagnosis to either relapse, death (if no relapse had been observed) or last follow-up for censored patients. Survival curves for OS and DFS with associated log-rank tests were generated using the Kaplan-Meier method ("Proc lifetest" function of SAS9.4 software). Survival median has been estimated using the inverted Kaplan-Meier method. Cox proportional hazards model was used to investigate confounding factors predicting for OS and DFS. The variables included in the model were: lymph node invasion status, tumour size and intrinsic breast cancer subtype. Variables sufficiently informed (less than 10% missing value in univariate analyses) and significant at a 10% level were included in a backward selection procedure to keep factors significant at a 5% level in the final multivariate model. Comparison of *NCL* expression levels between the three *NCL*-based groups of breast tumours with healthy donors was performed using non-parametric Kruskal-Wallis and Mann-Whitney tests

All *p*-values corresponded to two-tailed *p*-values. A p-value ≤ 0.05 was considered statistically significant. Statistical analyses were performed using either SAS v9.4 (SAS Institute) or GraphPad Prism v7.0a softwares (GraphPad Software, Inc). Graphical representations were depicted using GraphPad Prism software.

1.4. Transcriptomic Analyses

In addition to the clinical data and *NCL* expression extracted from the TCGA database, mRNA expression z-scores (RNA Seq V2 RSEM) for all genes were extracted from database cBioPortal for Cancer Genomics for the 661 identified breast tumours then divided in the three groups based on *NCL* expression ("low", "intermediate" and "high"). K-mean clustering has been done in two steps on these three *NCL* expression-based groups. First, k-mean clustering has been performed on each group ("low", "intermediate", "high") individually on both column wise ("samples") and row wise ("genes"). Number of clusters used were 5 for samples and 10 for genes. In-house R script (https://www.r-project.org/) was written for k-mean clustering using "K-mean" function of the "amap" package (https://cran.r-project.org/web/packages/amap/index.html) with the distance

calculation as Pearson correlation (parameters: method = "pearson", number of clusters = 10 for genes and 5 for samples). Second, these k-mean clustered on both samples and genes of the first step were concatenated as high, low and intermediate *NCL* groups. On these concatenated data, a second k-mean clustering was performed but only on genes with number of clusters as 10. Other parameters were equivalent to the ones used in the first step. Z-scores of the k-mean clusters issued from the second step were then arranged in cluster wise using the perl script. This clustered z-scores of the samples was used to plot an heatmap using the heatmap.2 function of the R packages gplots and RColorBrewer. Finally, gene ontology was performed on gene list issued from each identified clustered in this double k-mean clustering using DAVID tools (functional annotation clustering) [6].

2. Supplementary Figures

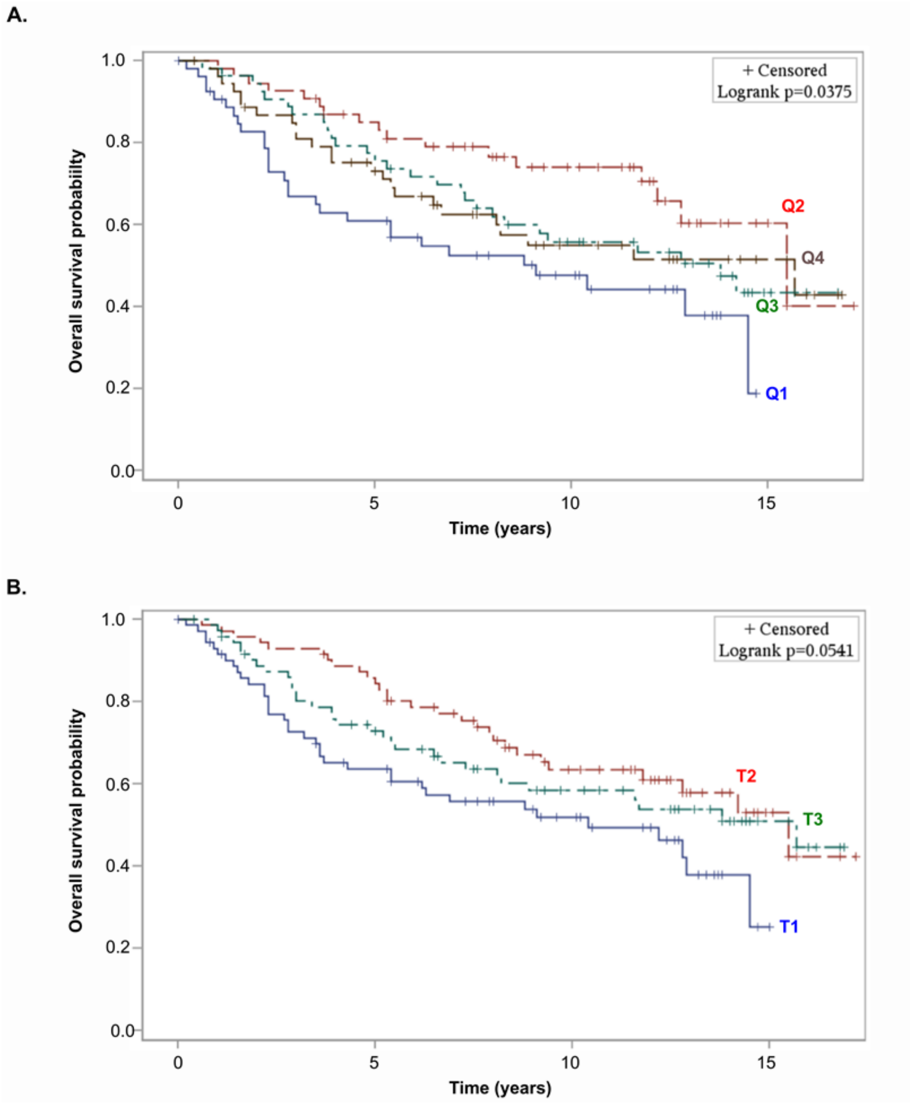

Figure S1. Cut-off determination for *NCL* expression using the Dundee series. Association between *NCL* mRNA expression levels and overall survival was analyzed in the Dundee series using Kaplan-Meier representation with two different cut-offs based either on quartile (**A**) or tercile (**B**) distribution. In the two conditions, patients carrying a tumour expressing either the lowest or the highest *NCL* mRNA levels had the poorest overall survival. Q: 4 groups based on quartile; T: 3 groups based on tercile. Log-rank p-value ≤ 0.05 was used to determine significant association.

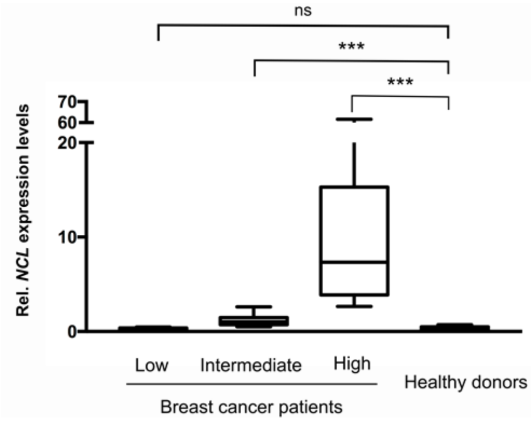

Figure S2. Comparison of *NCL* expression levels in tumours and healthy donors. Relative expression levels of *NCL* were compared in mammary tissues issued from healthy donors to that one of three groups of breast tumours identified as expressing different *NCL* expression levels (low, intermediate and high). *NCL* expression levels were significantly higher in "intermediate" and "high" groups, whereas no significant difference was observed between the "low" group and healthy donors. These data suggest that the "high" and "intermediate" groups, but not the "low" group, over-expressed *NCL* compared to healthy donors. ns: not significant. Kruskal-Wallis test *p*-value < 0.0001. ***: Mann-Whitney test *p*-value < 0.0001.

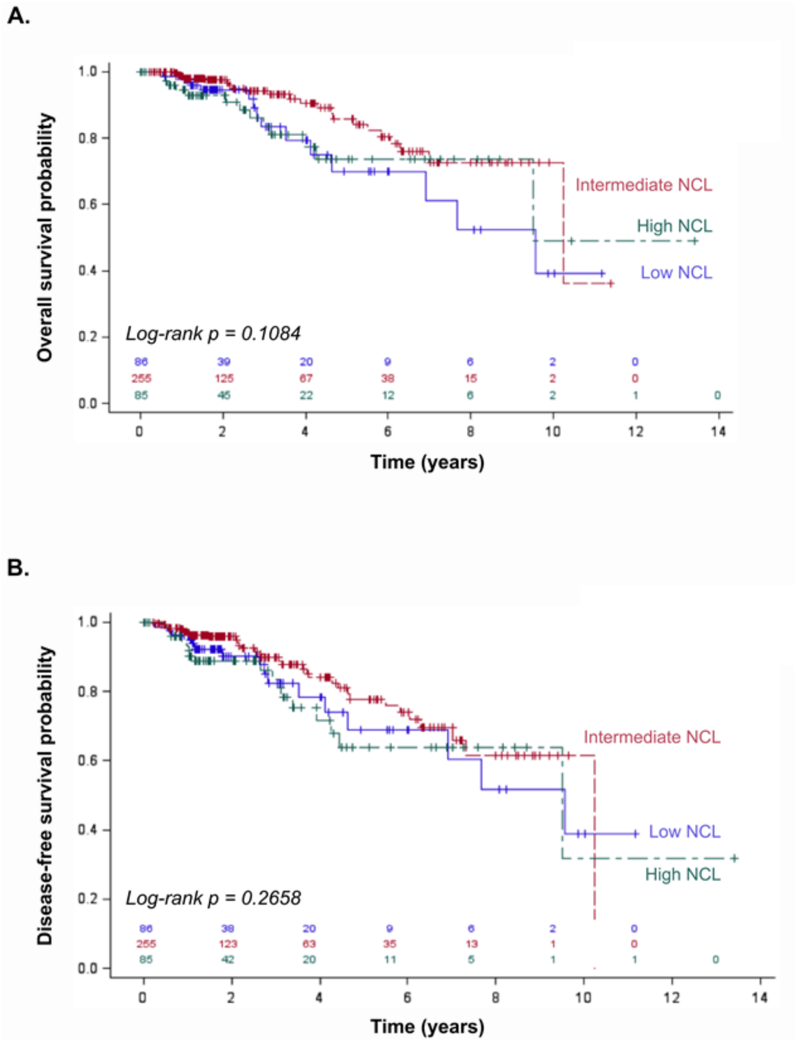

Figure S3. Association of *NCL* expression levels with survivals of breast cancer patients using TCGA series. Analyses of overall (**A**) and disease-free survivals (**B**) in all breast cancer patients. Using cut-off determined with the Dundee series, association between *NCL* expression levels and

survivals were analysed in the TCGA validation series. The curves' tendency suggests an association was observed between *NCL* expression levels and overall survival, while no association was observed in disease-free survival. Number of subjects at risk is indicated on the graph for the three *NCL* groups (low; intermediate; high). Log-rank p-value ≤ 0.05 was used to determine significant association.

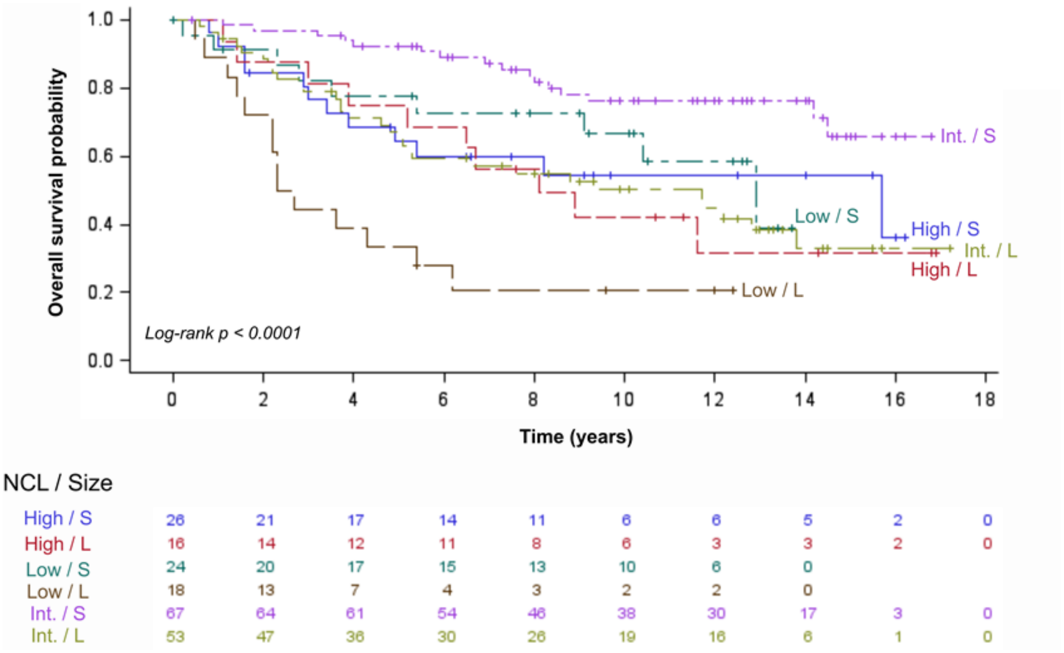

Figure S4. Discrimination of the most aggressive breast tumours with large size at diagnosis using *NCL* expression. Association between tumour size and *NCL* expression levels with overall survival was analysed in the Dundee series using Kaplan-Meier curves. Patients carrying large breast tumours (≥ 30 mm) and expressing low *NCL* expression levels presented a poorer overall survival compared to patients carrying large breast tumours but expressing either intermediate or high *NCL* expression levels. Number of subjects at risk is indicated on the graph for the six groups combining different *NCL* expression levels (low; inter.: intermediate; high) and tumour size (S: small, < 30mm; L: large, ≥ 30 mm). Log-rank p-value ≤ 0.05 was used to determine significant association.

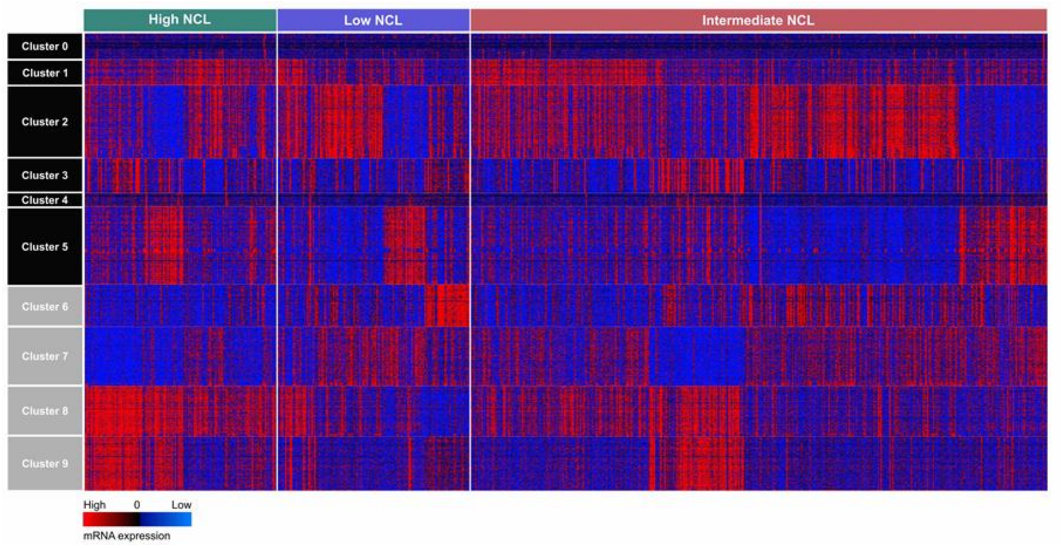

Figure S5. Comparison of gene expression profiles between the three *NCL* expressing groups of breast tumours in TCGA series. Heatmap was generated using transcriptome datasets extracted from the TCGA database for the 661 breast tumours. Columns correspond to breast tumour samples that have been grouped according to *NCL* expression (high, low and intermediate). Rows

correspond to genes that were assembled in ten clusters (from 0 to 9) using the k-means approach. Gene expression profiles between the low and high *NCL* expressing breast tumours showed difference in global mRNA levels in four clusters: 6, 7, 8 and 9 (grey). These data suggest that the *NCL* expressing breast tumours are different from each other. Gene expression scale is given at the bottom of the figure (Blue: low expression level; red: high expression level).

A.

	Category	Term	Gene number	% Gene	Fold Enrichment
	**FAC 1 - Enrichment Score: 26.262**				
	GOTERM_BP_FAT	GO:0006952~defense response	121	14,67	4,127
	GOTERM_BP_FAT	GO:0006954~inflammatory response	63	7,64	4,066
	GOTERM_BP_FAT	GO:0009611~response to wounding	74	8,97	2,928
	**FAC 2 - Enrichment Score: 23.070**				
Cluster 6 **Immunity**	UP_SEQ_FEATURE	disulfide bond	233	28,24	1,967
	SP_PIR_KEYWORDS	disulfide bond	238	28,85	1,945
	UP_SEQ_FEATURE	topological domain:Extracellular	226	27,39	1,978
	**FAC 3 - Enrichment Score: 15.981**				
	UP_SEQ_FEATURE	topological domain:Extracellular	226	27,39	1,978
	SP_PIR_KEYWORDS	glycoprotein	300	36,36	1,66
	UP_SEQ_FEATURE	glycosylation site:N-linked (GlcNAc...)	290	35,15	1,672

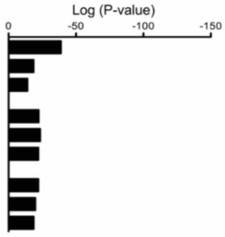

B.

	Category	Term	Gene number	% Gene	Fold Enrichment
	**FAC 1 - Enrichment Score: 33.587**				
	SP_PIR_KEYWORDS	glycoprotein	443	41,52	1,878
	UP_SEQ_FEATURE	glycosylation site:N-linked (GlcNAc...)	424	39,74	1,869
	SP_PIR_KEYWORDS	signal	349	32,71	1,965
	**FAC 2 - Enrichment Score: 21.215**				
Cluster 7 **Cell adhesion**	GOTERM_BP_FAT	GO:0007155~cell adhesion	112	10,50	2,682
	GOTERM_BP_FAT	GO:0022610~biological adhesion	112	10,50	2,678
	SP_PIR_KEYWORDS	cell adhesion	78	7,31	3,383
	**FAC 3 - Enrichment Score: 18.062**				
	SP_PIR_KEYWORDS	glycoprotein	443	41,52	1,878
	UP_SEQ_FEATURE	glycosylation site:N-linked (GlcNAc...)	424	39,74	1,869
	UP_SEQ_FEATURE	topological domain:Extracellular	246	23,06	1,647

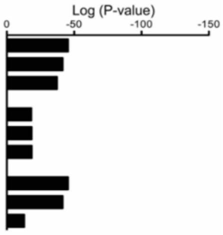

C.

	Category	Term	Gene number	% Gene	Fold Enrichment
	**FAC 1 - Enrichment Score: 60.061**				
	SP_PIR_KEYWORDS	zinc-finger	415	27,14	3,069
	UP_SEQ_FEATURE	zinc finger region:C2H2-type 7	204	13,34	5,714
	UP_SEQ_FEATURE	zinc finger region:C2H2-type 6	210	13,73	5,436
	**FAC 2 - Enrichment Score: 10.303**				
Cluster 8 **Transcription**	SP_PIR_KEYWORDS	ubl conjugation pathway	93	6,08	2,321
	GOTERM_BP_FAT	GO:0051603~proteolysis involved in cellular protein catabolic process	108	7,06	2,044
	GOTERM_BP_FAT	GO:0044257~cellular protein catabolic process	108	7,06	2,034
	**FAC 3 - Enrichment Score: 6.725**				
	INTERPRO	IPR001394:Peptidase C19, ubiquitin carboxyl-terminal hydrolase 2	26	1,70	5,029
	INTERPRO	IPR018200:Peptidase C19, ubiquitin carboxyl-terminal hydrolase 2, conserved site	25	1,64	4,999
	GOTERM_MF_FAT	GO:0004221~ubiquitin thiolesterase activity	27	1,77	3,974

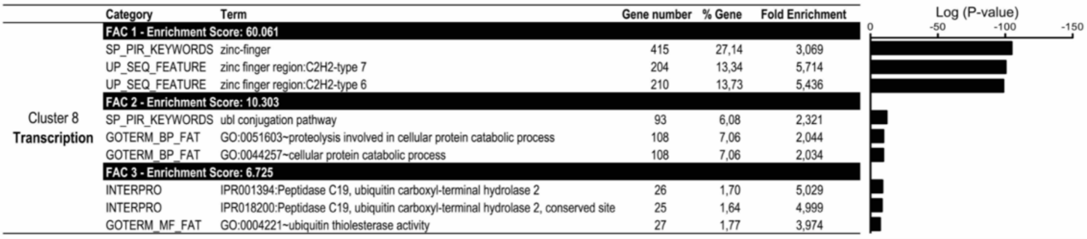

D.

	Category	Term	Gene number	% Gene	Fold Enrichment
	**FAC 1 - Enrichment Score: 16.482**				
	UP_SEQ_FEATURE	region of interest:Linker 12	34	2,77	8,419
	UP_SEQ_FEATURE	region of interest:Coil 2	34	2,77	8,419
	UP_SEQ_FEATURE	region of interest:Linker 1	35	2,85	7,924
	**FAC 2 - Enrichment Score: 14.207**				
Cluster 9 **Translation**	GOTERM_BP_FAT	GO:0007398~ectoderm development	60	4,88	4,414
	GOTERM_BP_FAT	GO:0008544~epidermis development	56	4,56	4,456
	SP_PIR_KEYWORDS	keratinization	23	1,87	9,156
	**FAC 3 - Enrichment Score: 7.469**				
	SP_PIR_KEYWORDS	ribonucleoprotein	60	4,88	3,424
	GOTERM_CC_FAT	GO:0030529~ribonucleoprotein complex	84	6,83	2,296
	SP_PIR_KEYWORDS	ribosomal protein	39	3,17	3,303

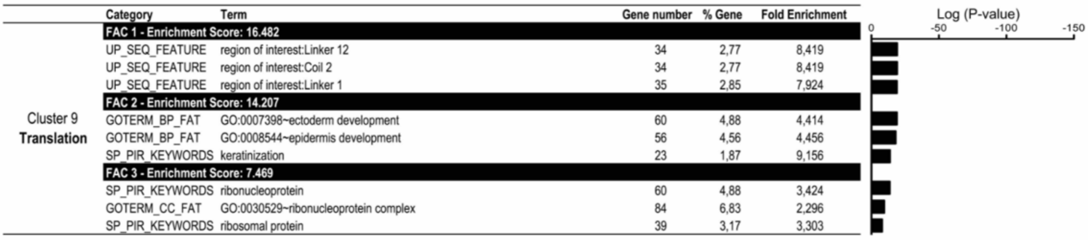

Figure S6. Gene ontology analysis on the clusters for which gene expression is different between low and high *NCL* expressing breast tumours. Gene ontology (GO) functional annotation clustering was performed using DAVID tools for annotation, visualization and integrated discovery. The GO analysis was restricted to the clusters 6 to 9 identified in Figure S5 that showed different expression profiles between low and high *NCL* expressing breast tumours. For each cluster, the top three functional annotation clusters (FAC) ordered by their enrichment score as well as the top three annotation terms are shown. Enrichment of genes involved in cell immunity was observed for the cluster 6 (**A**), in cell adhesion for cluster 7 (**B**), in transcription for cluster 8 (**C**) and in translation for cluster 9 (**D**). The low and high *NCL* expressing tumours express different gene profiling associated with distinct biological functions. For complete GO analysis see Supplementary table 4.

A

	Gene Ranking Order	
**Methods**	**Most stable gene**	**Least stable gene**
**Delta CT**	*GAPDH*	*ACTB*
**BestKeeper**	*ACTB*	*GAPDH*
**Normfinder**	*GAPDH*	*ACTB*
**GeNORM**	*ACTB/GAPDH*	
**Recommended comprehensive ranking**	*GAPDH*	*ACTB*

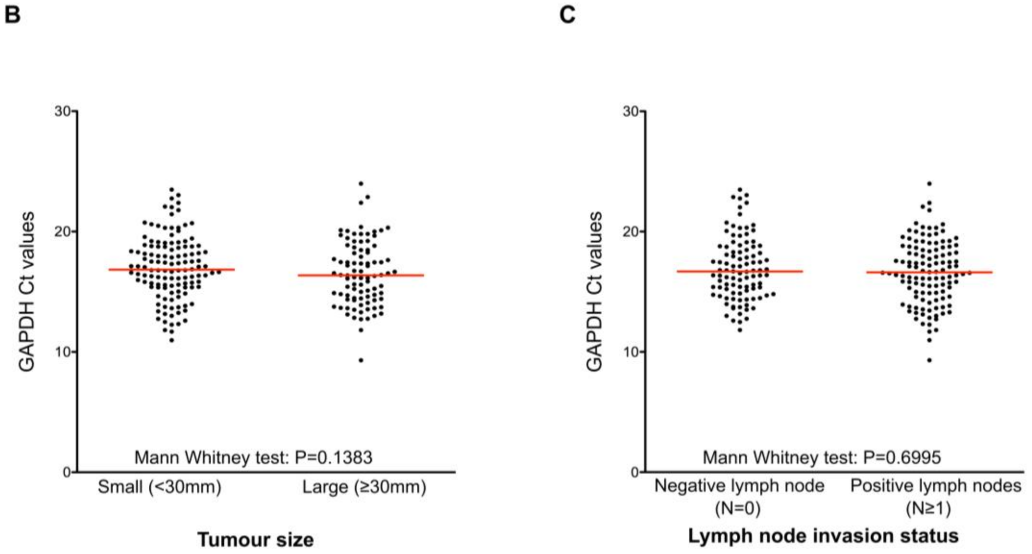

Figure S7. Determination of the most stable housekeeping gene in our study to normalize RT-qPCR analyses. (**A**) Stability ranking of *GAPDH* and *ACTB* genes in the 216 samples of Dundee series using RefFinder tool. *GAPDH* expression was determined as more stable than *ACTB* one. (**B**) Association of *GAPDH* expression stability with tumour size and lymph node invasion status. GAPDH expression was not significantly different in both small and large tumours or in lymph node non-invaded and invaded tumours.

3. Supplementary Tables

Table S1. Characteristics of patients issued from the TCGA dataset.

Characteristics	TCGA Series (*n* = 661)
**Age**	
Median	58
(min max)	(26–90)
**Tumour size**	
No data	1
≤20 mm	180 (27.3%)
>20 mm	480 (72.7%)
**ER status**	
No data	42
Negative	153 (24.7%)
Positive	466 (75.3%)
**PR status**	
No data	46
Negative	205 (33.3%)
Positive	410 (66.7%)
**HER2 status**	
No data	226
Negative	342 (78.6%)
Positive	93 (21.4%)
**Intrinsic breast cancer subtype**	
No data	235
ER+ PR+/– HER2–	265 (62.2%)
ER+/– PR+/– HER2+	93 (21.8%)
ER– PR– HER2–	68 (16.0%)
**Histological breast cancer subtype**	
No data	1
IDC	518 (78.5%)
ILC	101 (15.3%)
Others	41 (6.2%)

IDC: Infiltrating Ductal Carcinoma; ILC: Infiltrating Lobular Carcinoma.

Table S2. Nucleotide sequences of primers used in the study.

Gene Name	Forward (5′ To 3′)	Reverse (5′ To 3′)
NCL	CCA GAA CCA AAA TGG CAA AT	CTG ATT GCT CTG CCC TCA AT
GAPDH	AGC CAC ATC GCT CAG ACA C	GCC CAA TAC GAC CAA ATC C

Table S3. Univariate Cox regression models for survivals in Dundee series.

Variable		Overall Survival			Disease-Free Survival		
		HR	CI 95%	*p*-Value	HR	CI 95%	*p*-Value
NCL	Intermediate	1.00			1.00		
	Low	2.13	(1.30–3.48)		2.20	(1.37–3.52)	
	High	1.49	(0.90–2.46)	0.0083	1.60	(1.00–2.58)	0.0030
Tumour size	<30 mm	1.00			1.00		
	≥30 mm	2.46	(1.61–3.73)	<0.0001	2.13	(1.43–3.17)	0.0002
Invaded lymph node	*n* = 0	1.00			1.00		
	n ≥ 1	1.77	(1.16–2.70)	0.0079	1.82	(1.22–2.72)	0.0034
Intrinsic breast cancer subtype	ER+ PR+/– HER2–	1.00			1.00		
	ER+/– PR+/– HER2+	1.31	(0.80–2.13)		1.31	(0.82–2.07)	
	ER– PR– HER2–	1.93	(1.12–3.32)	0.0585	1.63	(0.96–2.77)	0.1789

HR: Hazard Ratio; CI 95%: 95% Confidence Interval.

Table S4. Full gene ontology analysis. It can be found in the appendix.

References

1. Bourdon, J.-C.; Khoury, M.P.; Diot, A.; Baker, L.; Fernandes, K.; Aoubala, M.; Quinlan, P.; Purdie, C.A.; Jordan, L.B.; Prats, A.-C.; et al. p53 mutant breast cancer patients expressing p53γ have as good a prognosis as wild-type p53 breast cancer patients. *Breast Cancer Res.* **2011**, *13*, R7.
2. Ciriello, G.; Gatza, M.L.; Beck, A.H.; Wilkerson, M.D.; Rhie, S.K.; Pastore, A.; Zhang, H.; McLellan, M.; Yau, C.; Kandoth, C.; et al. TCGA Research Network; Perou, C.M. Comprehensive Molecular Portraits of Invasive Lobular Breast Cancer. *Cell* **2015**, *163*, 506–519.
3. Maire, V.; Baldeyron, C.; Richardson, M.; Tesson, B.; Vincent-Salomon, A.; Gravier, E.; Marty-Prouvost, B.; De Koning, L.; Rigaill, G.; Dumont, A.; et al. TTK/hMPS1 is an attractive therapeutic target for triple-negative breast cancer. *PLoS ONE* **2013**, *8*, e63712.
4. Marcel, V.; Catez, F.; Berger, C.M.; Perrial, E.; Plesa, A.; Thomas, X.; Mattei, E.; Hayette, S.; Saintigny, P.; Bouvet, P.; et al. Expression Profiling of Ribosome Biogenesis Factors Reveals Nucleolin as a Novel Potential Marker to Predict Outcome in AML Patients. *PLoS ONE* **2017**, *12*, e0170160.
5. Xie, F.; Xiao, P.; Chen, D.; Xu, L.; Zhang, B. miRDeepFinder: A miRNA analysis tool for deep sequencing of plant small RNAs. *Plant Mol. Biol.* **2012**, doi:10.1007/s11103-012-9885-2
6. Huang, D.W.; Sherman B.T.; Lempicki, R.A. Systematic and integrative analysis of large gene lists using DAVID bioinformatics resources. *Nat. Protoc.* **2009**, *4*, 44–57.

© 2018 by the authors. Licensee MDPI, Basel, Switzerland. This article is an open access article distributed under the terms and conditions of the Creative Commons Attribution (CC BY) license (http://creativecommons.org/licenses/by/4.0/).
