# Supplementary material for: Druggable Nucleolin Identifies Breast Tumours Associated with Poor Prognosis That Exhibit Different Biological Processes
Source: Cancers (Basel). 2018 Oct 22;10(10):390. doi: 10.3390/cancers10100390 (PMC6210205; doi:10.3390/cancers10100390)
Supplement: Supplementary file 1 [file cancers-10-00390-s001.zip › Table S4.pdf]

Annotation Cluster 1
Category
UP_SEQ_FEATURE
UP_SEQ_FEATURE
UP_SEQ_FEATURE
UP_SEQ_FEATURE
UP_SEQ_FEATURE
UP_SEQ_FEATURE
UP_SEQ_FEATURE
SP_PIR_KEYWORDS
UP_SEQ_FEATURE
INTERPRO
INTERPRO
PIR_SUPERFAMILY
INTERPRO
INTERPRO
GOTERM_MF_FAT
UP_SEQ_FEATURE
SP_PIR_KEYWORDS
GOTERM_CC_FAT
GOTERM_CC_FAT
GOTERM_CC_FAT
INTERPRO

Annotation Cluster 2
Category
GOTERM_BP_FAT
GOTERM_BP_FAT
SP_PIR_KEYWORDS
GOTERM_BP_FAT
GOTERM_BP_FAT
GOTERM_BP_FAT
INTERPRO
GOTERM_BP_FAT
GOTERM_BP_FAT
GOTERM_CC_FAT

Annotation Cluster 3
Category
SP_PIR_KEYWORDS
GOTERM_CC_FAT
SP_PIR_KEYWORDS
GOTERM_MF_FAT
GOTERM_CC_FAT
GOTERM_CC_FAT
GOTERM_CC_FAT

GOTERM_BP_FAT
GOTERM_CC_FAT
GOTERM_CC_FAT
GOTERM_CC_FAT
GOTERM_CC_FAT

Annotation Cluster 4
Category
GOTERM_CC_FAT
GOTERM_BP_FAT
GOTERM_MF_FAT

Annotation Cluster 5
Category
KEGG_PATHWAY
SP_PIR_KEYWORDS
GOTERM_CC_FAT
GOTERM_BP_FAT
GOTERM_BP_FAT
GOTERM_BP_FAT
GOTERM_BP_FAT
INTERPRO
GOTERM_BP_FAT
SP_PIR_KEYWORDS
GOTERM_BP_FAT
GOTERM_MF_FAT
GOTERM_MF_FAT
GOTERM_BP_FAT
SP_PIR_KEYWORDS
GOTERM_BP_FAT
GOTERM_CC_FAT
GOTERM_BP_FAT
GOTERM_BP_FAT
GOTERM_BP_FAT
GOTERM_BP_FAT
GOTERM_BP_FAT
INTERPRO
GOTERM_BP_FAT
GOTERM_BP_FAT
INTERPRO
GOTERM_BP_FAT
GOTERM_BP_FAT
SP_PIR_KEYWORDS
INTERPRO
GOTERM_BP_FAT
GOTERM_BP_FAT
GOTERM_BP_FAT
GOTERM_BP_FAT

GOTERM_BP_FAT
GOTERM_BP_FAT
GOTERM_BP_FAT
GOTERM_BP_FAT
GOTERM_BP_FAT
GOTERM_BP_FAT
GOTERM_BP_FAT

Annotation Cluster 6
Category
INTERPRO
INTERPRO
UP_SEQ_FEATURE
PIR_SUPERFAMILY
UP_SEQ_FEATURE
SP_PIR_KEYWORDS
UP_SEQ_FEATURE
UP_SEQ_FEATURE
INTERPRO
INTERPRO
INTERPRO
INTERPRO

Annotation Cluster 7
Category
BIOCARTA
KEGG_PATHWAY
SMART
SP_PIR_KEYWORDS
INTERPRO
INTERPRO
GOTERM_CC_FAT
SP_PIR_KEYWORDS
GOTERM_BP_FAT
GOTERM_BP_FAT
SP_PIR_KEYWORDS
GOTERM_BP_FAT
GOTERM_CC_FAT
GOTERM_BP_FAT
GOTERM_BP_FAT
GOTERM_BP_FAT
GOTERM_BP_FAT
SP_PIR_KEYWORDS
GOTERM_BP_FAT

Annotation Cluster 8
Category
INTERPRO

SMART
UP_SEQ_FEATURE
INTERPRO
INTERPRO
PIR_SUPERFAMILY
SP_PIR_KEYWORDS

Annotation Cluster 9
Category
INTERPRO
GOTERM_MF_FAT
SMART
GOTERM_MF_FAT
INTERPRO
SP_PIR_KEYWORDS
GOTERM_MF_FAT
SP_PIR_KEYWORDS
INTERPRO
UP_SEQ_FEATURE
UP_SEQ_FEATURE
GOTERM_MF_FAT
SMART
PIR_SUPERFAMILY
INTERPRO
UP_SEQ_FEATURE
UP_SEQ_FEATURE

Annotation Cluster 10
Category
SMART
SP_PIR_KEYWORDS
UP_SEQ_FEATURE
INTERPRO
INTERPRO
GOTERM_MF_FAT
INTERPRO
GOTERM_MF_FAT
UP_SEQ_FEATURE
GOTERM_MF_FAT
GOTERM_MF_FAT
GOTERM_MF_FAT
SP_PIR_KEYWORDS
GOTERM_MF_FAT

Annotation Cluster 11
Category
SP_PIR_KEYWORDS
SP_PIR_KEYWORDS

GOTERM_CC_FAT

Annotation Cluster 12
Category
SP_PIR_KEYWORDS
UP_SEQ_FEATURE
INTERPRO

Annotation Cluster 13
Category
GOTERM_CC_FAT
GOTERM_CC_FAT
GOTERM_CC_FAT
GOTERM_CC_FAT
GOTERM_CC_FAT

Annotation Cluster 14
Category
INTERPRO
INTERPRO
SP_PIR_KEYWORDS
SP_PIR_KEYWORDS
INTERPRO
UP_SEQ_FEATURE
UP_SEQ_FEATURE
GOTERM_MF_FAT
GOTERM_MF_FAT
GOTERM_MF_FAT
GOTERM_MF_FAT
GOTERM_BP_FAT
INTERPRO
UP_SEQ_FEATURE
PIR_SUPERFAMILY
INTERPRO
UP_SEQ_FEATURE
UP_SEQ_FEATURE
INTERPRO
GOTERM_CC_FAT
INTERPRO
SP_PIR_KEYWORDS

Annotation Cluster 15
Category
SP_PIR_KEYWORDS
GOTERM_BP_FAT
GOTERM_BP_FAT
KEGG_PATHWAY
GOTERM_BP_FAT

GOTERM_BP_FAT
GOTERM_BP_FAT
GOTERM_BP_FAT
GOTERM_BP_FAT
GOTERM_BP_FAT
GOTERM_MF_FAT

Annotation Cluster 16
Category
SP_PIR_KEYWORDS
GOTERM_CC_FAT
SP_PIR_KEYWORDS
KEGG_PATHWAY
UP_SEQ_FEATURE
GOTERM_CC_FAT
GOTERM_CC_FAT
GOTERM_BP_FAT
GOTERM_CC_FAT
GOTERM_CC_FAT
GOTERM_CC_FAT
SP_PIR_KEYWORDS
GOTERM_CC_FAT
GOTERM_CC_FAT
GOTERM_CC_FAT
GOTERM_BP_FAT
SP_PIR_KEYWORDS
KEGG_PATHWAY
GOTERM_CC_FAT
GOTERM_BP_FAT
GOTERM_CC_FAT
GOTERM_CC_FAT
GOTERM_CC_FAT
SP_PIR_KEYWORDS
GOTERM_BP_FAT
GOTERM_BP_FAT
KEGG_PATHWAY
GOTERM_BP_FAT
GOTERM_MF_FAT
GOTERM_BP_FAT

Annotation Cluster 17
Category
INTERPRO
INTERPRO
INTERPRO
INTERPRO
PIR_SUPERFAMILY
GOTERM_BP_FAT

INTERPRO
GOTERM_BP_FAT

Annotation Cluster 18
Category
GOTERM_MF_FAT
SP_PIR_KEYWORDS
GOTERM_MF_FAT
GOTERM_MF_FAT
GOTERM_MF_FAT
GOTERM_MF_FAT
INTERPRO

Annotation Cluster 19
Category
GOTERM_MF_FAT
UP_SEQ_FEATURE
UP_SEQ_FEATURE
INTERPRO
SP_PIR_KEYWORDS
SP_PIR_KEYWORDS
INTERPRO
INTERPRO
PIR_SUPERFAMILY
SP_PIR_KEYWORDS
SP_PIR_KEYWORDS
SP_PIR_KEYWORDS
UP_SEQ_FEATURE
UP_SEQ_FEATURE

Annotation Cluster 20
Category
SMART
INTERPRO
INTERPRO
INTERPRO
PIR_SUPERFAMILY
GOTERM_BP_FAT

Annotation Cluster 21
Category
GOTERM_BP_FAT
GOTERM_BP_FAT
GOTERM_BP_FAT
GOTERM_BP_FAT
GOTERM_BP_FAT

Annotation Cluster 22

Category
KEGG_PATHWAY
GOTERM_BP_FAT
GOTERM_BP_FAT
GOTERM_BP_FAT
GOTERM_BP_FAT
GOTERM_BP_FAT
GOTERM_BP_FAT
GOTERM_BP_FAT
GOTERM_BP_FAT
GOTERM_BP_FAT
GOTERM_BP_FAT
GOTERM_BP_FAT
GOTERM_BP_FAT
SP_PIR_KEYWORDS
GOTERM_BP_FAT
GOTERM_BP_FAT
GOTERM_BP_FAT
GOTERM_BP_FAT
GOTERM_BP_FAT
GOTERM_BP_FAT
GOTERM_BP_FAT
GOTERM_BP_FAT

Annotation Cluster 23
Category
UP_SEQ_FEATURE
UP_SEQ_FEATURE
PIR_SUPERFAMILY

Annotation Cluster 24
Category
BIOCARTA
BIOCARTA
BIOCARTA

Annotation Cluster 25
Category
GOTERM_MF_FAT
GOTERM_BP_FAT
GOTERM_MF_FAT

Annotation Cluster 26
Category
SP_PIR_KEYWORDS
GOTERM_CC_FAT
GOTERM_CC_FAT
GOTERM_CC_FAT

Annotation Cluster 27
Category
SMART
INTERPRO
INTERPRO

Annotation Cluster 28
Category
INTERPRO
SMART
UP_SEQ_FEATURE
UP_SEQ_FEATURE
UP_SEQ_FEATURE
SMART
INTERPRO
INTERPRO
PIR_SUPERFAMILY
INTERPRO
INTERPRO

Annotation Cluster 29
Category
INTERPRO
COG_ONTOLOGY
INTERPRO

Annotation Cluster 30
Category
INTERPRO
INTERPRO
INTERPRO

Annotation Cluster 31
Category
GOTERM_CC_FAT
GOTERM_CC_FAT
GOTERM_CC_FAT

Annotation Cluster 32
Category
PIR_SUPERFAMILY
UP_SEQ_FEATURE
INTERPRO

Annotation Cluster 33
Category
INTERPRO

SP_PIR_KEYWORDS
GOTERM_MF_FAT
INTERPRO
PIR_SUPERFAMILY

Annotation Cluster 34
Category
GOTERM_BP_FAT
GOTERM_BP_FAT
GOTERM_BP_FAT
GOTERM_BP_FAT

Annotation Cluster 35
Category
GOTERM_BP_FAT
GOTERM_MF_FAT
GOTERM_MF_FAT

Enrichment Score: 16.481688970685838
Term
region of interest:Linker 12
region of interest:Coil 2
region of interest:Linker 1
region of interest:Coil 1B
region of interest:Coil 1A
region of interest:Rod
region of interest:Head
Intermediate filament
region of interest:Tail
IPR016044:Filament
IPR018039:Intermediate filament protein, conserved site
PIRSF002282:cytoskeletal keratin
IPR001664:Intermediate filament protein
IPR003054:Type II keratin
GO:0005198~structural molecule activity
site:Stutter
keratin
GO:0005882~intermediate filament
GO:0045095~keratin filament
GO:0045111~intermediate filament cytoskeleton
IPR002957:Keratin, type I

Enrichment Score: 14.207278284320084
Term
GO:0007398~ectoderm development
GO:0008544~epidermis development
keratinization
GO:0031424~keratinization
GO:0030216~keratinocyte differentiation
GO:0009913~epidermal cell differentiation
IPR003267:Small proline-rich
GO:0030855~epithelial cell differentiation
GO:0060429~epithelium development
GO:0001533~cornified envelope

Enrichment Score: 7.4691752796059445
Term
ribonucleoprotein
GO:0030529~ribonucleoprotein complex
ribosomal protein
GO:0003735~structural constituent of ribosome
GO:0005840~ribosome
GO:0000313~organellar ribosome
GO:0005761~mitochondrial ribosome

GO:0006412~translation
GO:0033279~ribosomal subunit
GO:0015935~small ribosomal subunit
GO:0000314~organellar small ribosomal subunit
GO:0005763~mitochondrial small ribosomal subunit

Enrichment Score: 6.406599892920229
Term
GO:0030529~ribonucleoprotein complex
GO:0006396~RNA processing
GO:0003723~RNA binding

Enrichment Score: 5.102192781675423
Term
hsa03050:Proteasome
proteasome
GO:0000502~proteasome complex
GO:0031145~anaphase-promoting complex-dependent proteasomal ubiquiti
GO:0051436~negative regulation of ubiquitin-protein ligase activity
GO:0051444~negative regulation of ubiquitin-protein ligase activity
GO:0051352~negative regulation of ligase activity
IPR001353:Proteasome, subunit alpha/beta
GO:0051437~positive regulation of ubiquitin-protein ligase activity
threonine protease
GO:0051443~positive regulation of ubiquitin-protein ligase activity
GO:0070003~threonine-type peptidase activity
GO:0004298~threonine-type endopeptidase activity
GO:0051439~regulation of ubiquitin-protein ligase activity during mi
proteinase
GO:0051351~positive regulation of ligase activity
GO:0005839~proteasome core complex
GO:0031397~negative regulation of protein ubiquitination
GO:0031398~positive regulation of protein ubiquitination
GO:0051438~regulation of ubiquitin-protein ligase activity
GO:0051340~regulation of ligase activity
GO:0031396~regulation of protein ubiquitination
IPR001353:Proteasome, alpha and beta subunits
GO:0043161~proteasomal ubiquitin-dependent protein catabolic process
GO:0010498~proteasomal protein catabolic process
IPR016050:Proteasome, beta-type subunit, conserved site
GO:0000278~mitotic cell cycle
GO:0031400~negative regulation of protein modification process
protein degradation
IPR000426:Proteasome, alpha-subunit, conserved site
GO:0051248~negative regulation of protein metabolic process
GO:0022402~cell cycle process
GO:0031401~positive regulation of protein modification process
GO:0032269~negative regulation of cellular protein metabolic process

GO:0007049~cell cycle
GO:0044092~negative regulation of molecular function
GO:0043086~negative regulation of catalytic activity
GO:0006511~ubiquitin-dependent protein catabolic process
GO:0010605~negative regulation of macromolecule metabolic process
GO:0032270~positive regulation of cellular protein metabolic process
GO:0031399~regulation of protein modification process

Enrichment Score: 4.462221191827637
Term
IPR013787:S100/CaBP-9k-type, calcium binding, subdomain
IPR001751:S100/CaBP-9k-type, calcium binding
calcium-binding region:2; high affinity
PIRSF002353:S-100 protein
calcium-binding region:1; low affinity
EF hand
domain:EF-hand 2
domain:EF-hand 1
IPR011992:EF-Hand type
IPR018248:EF hand
IPR018249:EF-HAND 2
IPR018247:EF-HAND 1

Enrichment Score: 4.187211849721411
Term
h_smPathway:Spliceosomal Assembly
hsa03040:Spliceosome
SM00651:Sm
mrna splicing
IPR006649:Like-Sm ribonucleoprotein, eukaryotic and archaea-type, co
IPR001163:Like-Sm ribonucleoprotein, core
GO:0030532~small nuclear ribonucleoprotein complex
mrna processing
GO:0008380~RNA splicing
GO:0006396~RNA processing
Spliceosome
GO:0006397~mRNA processing
GO:0005681~spliceosome
GO:0000375~RNA splicing, via transesterification reactions
GO:0000377~RNA splicing, via transesterification reactions with bulg
GO:0000398~nuclear mRNA splicing, via spliceosome
GO:0016071~mRNA metabolic process
rna-binding
GO:0000387~spliceosomal snRNP biogenesis

Enrichment Score: 4.0753555027506945
Term
IPR018363:CD59 antigen, conserved site

SM00134:LU
domain:UPAR/Ly6
IPR016054:Ly-6 antigen / uPA receptor -like
IPR001526:CD59 antigen
PIRSF002021:Ly-6-like protein
gpi-anchor

Enrichment Score: 3.3705598797631997
Term
IPR018069:Whey acidic protein, 4-disulphide core, conserved site
GO:0004867~serine-type endopeptidase inhibitor activity
SM00217:WAP
GO:0030414~peptidase inhibitor activity
IPR008197:Whey acidic protein, 4-disulphide core
Serine protease inhibitor
GO:0004866~endopeptidase inhibitor activity
protease inhibitor
IPR015874:4-disulphide core
site:Reactive bond
domain:WAP
GO:0004857~enzyme inhibitor activity
SM00093:SERPIN
PIRSF001630:serpin
IPR000215:Protease inhibitor I4, serpin
domain:WAP 1
domain:WAP 2

Enrichment Score: 3.2924412815974016
Term
SM00020:Tryp_SPc
Serine protease
domain:Peptidase S1
IPR001314:Peptidase S1A, chymotrypsin
IPR018114:Peptidase S1/S6, chymotrypsin/Hap, active site
GO:0004252~serine-type endopeptidase activity
IPR001254:Peptidase S1 and S6, chymotrypsin/Hap
GO:0008236~serine-type peptidase activity
active site:Charge relay system
GO:0017171~serine hydrolase activity
GO:0004175~endopeptidase activity
GO:0008233~peptidase activity
Protease
GO:0070011~peptidase activity, acting on L-amino acid peptides

Enrichment Score: 2.7498696018301723
Term
Secreted
signal

GO:0005576~extracellular region

Enrichment Score: 2.6645404281912803
Term
tumor antigen
domain:MAGE
IPR002190:MAGE protein

Enrichment Score: 2.5720541786430298
Term
GO:0070013~intracellular organelle lumen
GO:0031974~membrane-enclosed lumen
GO:0043233~organelle lumen
GO:0005730~nucleolus
GO:0031981~nuclear lumen

Enrichment Score: 2.419260644222206
Term
IPR017936:Thioredoxin-like
IPR006662:Thioredoxin-like subdomain
redox-active disulfide
Redox-active center
IPR017937:Thioredoxin, conserved site
domain:Thioredoxin 2
domain:Thioredoxin 1
GO:0016864~intramolecular oxidoreductase activity, transposing S-S b[illegible]
GO:0003756~protein disulfide isomerase activity
GO:0016860~intramolecular oxidoreductase activity
GO:0016862~intramolecular oxidoreductase activity, interconverting k[illegible]
GO:0045454~cell redox homeostasis
IPR005788:Disulphide isomerase
site:Contributes to redox potential value
PIRSF001487:protein disulfide-isomerase
IPR012335:Thioredoxin fold
site:Lowers pKa of C-terminal Cys of second active site
site:Lowers pKa of C-terminal Cys of first active site
IPR005792:Protein disulphide isomerase
GO:0005788~endoplasmic reticulum lumen
IPR013766:Thioredoxin domain
intramolecular oxidoreductase

Enrichment Score: 2.379794611005659
Term
tricarboxylic acid cycle
GO:0009060~aerobic respiration
GO:0045333~cellular respiration
hsa00020:Citrate cycle (TCA cycle)
GO:0006099~tricarboxylic acid cycle

GO:0046356~acetyl-CoA catabolic process
GO:0006084~acetyl-CoA metabolic process
GO:0051187~cofactor catabolic process
GO:0009109~coenzyme catabolic process
GO:0015980~energy derivation by oxidation of organic compounds
GO:0000104~succinate dehydrogenase activity

Enrichment Score: 2.3764889467219867
Term
mitochondrion
GO:0044429~mitochondrial part
transit peptide
hsa05012:Parkinson's disease
transit peptide:Mitochondrion
GO:0005759~mitochondrial matrix
GO:0031980~mitochondrial lumen
GO:0045333~cellular respiration
GO:0005739~mitochondrion
GO:0031967~organelle envelope
GO:0031975~envelope
mitochondrion inner membrane
GO:0031966~mitochondrial membrane
GO:0005740~mitochondrial envelope
GO:0044455~mitochondrial membrane part
GO:0015980~energy derivation by oxidation of organic compounds
electron transport
hsa05016:Huntington's disease
GO:0019866~organelle inner membrane
GO:0022904~respiratory electron transport chain
GO:0005746~mitochondrial respiratory chain
GO:0070469~respiratory chain
GO:0005743~mitochondrial inner membrane
respiratory chain
GO:0042775~mitochondrial ATP synthesis coupled electron transport
GO:0042773~ATP synthesis coupled electron transport
hsa00190:Oxidative phosphorylation
GO:0006091~generation of precursor metabolites and energy
GO:0016655~oxidoreductase activity, acting on NADH or NADPH, quinone
GO:0022900~electron transport chain

Enrichment Score: 2.3304572277620172
Term
IPR003008:Tubulin/FtsZ, GTPase domain
IPR018316:Tubulin/FtsZ, 2-layer sandwich domain
IPR017975:Tubulin, conserved site
IPR000217:Tubulin
PIRSF002306:tubulin
GO:0051258~protein polymerization

IPR002452:Alpha tubulin
GO:0043623~cellular protein complex assembly

Enrichment Score: 2.259829326076668
Term
GO:0015293~symporter activity
Symport
GO:0015370~solute:sodium symporter activity
GO:0015294~solute:cation symporter activity
GO:0005326~neurotransmitter transporter activity
GO:0005328~neurotransmitter:sodium symporter activity
IPR000175:Sodium:neurotransmitter symporter

Enrichment Score: 2.1646931511556664
Term
GO:0046870~cadmium ion binding
metal ion-binding site:Divalent metal cation; cluster B
metal ion-binding site:Divalent metal cation; cluster A
IPR018064:Metallothionein, vertebrate, metal binding site
metal-thiolate cluster
metal binding
IPR003019:Metallothionein superfamily, eukaryotic
IPR000006:Metallothionein, vertebrate
PIRSF002564:metallothionein
copper
chelation
cadmium
region of interest:Beta
region of interest:Alpha

Enrichment Score: 2.148913924442017
Term
SM00097:WNT1
IPR005817:Wnt superfamily
IPR005816:Secreted growth factor Wnt protein
IPR018161:Secreted growth factor Wnt protein, conserved site
PIRSF001784:int-1 transforming protein
GO:0007223~Wnt receptor signaling pathway, calcium modulating pathwa

Enrichment Score: 2.146428276323983
Term
GO:0022404~molting cycle process
GO:0001942~hair follicle development
GO:0022405~hair cycle process
GO:0042633~hair cycle
GO:0042303~molting cycle

Enrichment Score: 2.08993132746172

Term
hsa00030:Pentose phosphate pathway
GO:0016052~carbohydrate catabolic process
GO:0006007~glucose catabolic process
GO:0019748~secondary metabolic process
GO:0046365~monosaccharide catabolic process
GO:0044275~cellular carbohydrate catabolic process
GO:0046496~nicotinamide nucleotide metabolic process
GO:0006769~nicotinamide metabolic process
GO:0009820~alkaloid metabolic process
GO:0019320~hexose catabolic process
GO:0019362~pyridine nucleotide metabolic process
GO:0006006~glucose metabolic process
GO:0046164~alcohol catabolic process
pentose shunt
GO:0006098~pentose-phosphate shunt
GO:0006739~NADP metabolic process
GO:0019318~hexose metabolic process
GO:0006733~oxidoreduction coenzyme metabolic process
GO:0019321~pentose metabolic process
GO:0043603~cellular amide metabolic process
GO:0006096~glycolysis
GO:0005996~monosaccharide metabolic process

Enrichment Score: 2.0122183380477385
Term
region of interest:MHC class I alpha-2 like
region of interest:MHC class I alpha-1 like
PIRSF037807:retinoic acid early inducible protein 1

Enrichment Score: 2.00986841033491
Term
h_ranPathway:Cycling of Ran in nucleocytoplasmic transport
h_npcPathway:Mechanism of Protein Import into the Nucleus
h_ranMSpathway:Role of Ran in mitotic spindle regulation

Enrichment Score: 1.8479790211462275
Term
GO:0016866~intramolecular transferase activity
GO:0001522~pseudouridine synthesis
GO:0009982~pseudouridine synthase activity

Enrichment Score: 1.8424748512622717
Term
mitochondrion outer membrane
GO:0005741~mitochondrial outer membrane
GO:0031968~organelle outer membrane
GO:0019867~outer membrane

Enrichment Score: 1.7868766291085558
Term
SM00212:UBCc
IPR000608:Ubiquitin-conjugating enzyme, E2
IPR016135:Ubiquitin-conjugating enzyme/RWD-like

Enrichment Score: 1.7725296285956527
Term
IPR003879:Butyrophylin-like
SM00336:BBOX
zinc finger region:B box-type
zinc finger region:RING-type
domain:B30.2/SPRY
SM00589:PRY
IPR000315:Zinc finger, B-box
IPR001870:B302 (SPRY)-like
PIRSF001733:tripartite motif-containing protein
IPR006574:SPRY-associated
IPR003877:SPla/RYanodine receptor SPRY

Enrichment Score: 1.7617183849028941
Term
IPR001125:Recoverin
Signal transduction mechanisms / Cytoskeleton / Cell division and ch
IPR018248:EF hand

Enrichment Score: 1.7250058816775624
Term
IPR000566:Lipocalin-related protein and Bos/Can/Equ allergen
IPR012674:Calycin
IPR002345:Lipocalin

Enrichment Score: 1.6898436338801728
Term
GO:0015934~large ribosomal subunit
GO:0000315~organellar large ribosomal subunit
GO:0005762~mitochondrial large ribosomal subunit

Enrichment Score: 1.6308236999321186
Term
PIRSF001467:peptidylprolyl isomerase
domain:PPIase cyclophilin-type
IPR002130:Peptidyl-prolyl cis-trans isomerase, cyclophilin-type

Enrichment Score: 1.6245617340384846
Term
IPR000866:Alkyl hydroperoxide reductase/ Thiol specific antioxidant/

antioxidant
GO:0016209~antioxidant activity
IPR019479:Peroxiredoxin, C-terminal
PIRSF000239:alkyl hydroperoxide reductase C22 protein

Enrichment Score: 1.5736127165604337
Term
GO:0022613~ribonucleoprotein complex biogenesis
GO:0034660~ncRNA metabolic process
GO:0042254~ribosome biogenesis
GO:0006364~rRNA processing

Enrichment Score: 1.5339722611672193
Term
GO:0000041~transition metal ion transport
GO:0046915~transition metal ion transmembrane transporter activity
GO:0005385~zinc ion transmembrane transporter activity

Count	%	PValue
34	2.7664768	4.0201259
34	2.7664768	4.0201259
35	2.8478437	1.1053562
35	2.8478437	1.1053562
35	2.8478437	1.1053562
35	2.8478437	1.9906582
35	2.8478437	6.2183468
35	2.8478437	7.6055862
34	2.7664768	2.2874038
33	2.6851098	1.8927981
33	2.6851098	1.8927981
32	2.6037428	2.0352980
32	2.6037428	3.5097657
19	1.5459723	3.1533749
96	7.8112286	1.1976644
19	1.5459723	9.1772368
33	2.6851098	2.5771274
37	3.0105777	1.7016427
24	1.9528071	2.9095418
37	3.0105777	3.0626252
12	0.9764035	4.7047659

Count	%	PValue
60	4.8820179	5.1121634
56	4.5565500	1.0030547
23	1.8714401	1.0611751
24	1.9528071	2.8341785
29	2.3596419	3.8295508
30	2.4410089	7.1734982
17	1.3832384	1.2522632
35	2.8478437	2.3996869
40	3.2546786	7.3200851
11	0.8950366	9.0746166

Count	%	PValue
60	4.8820179	6.5395016
84	6.8348250	8.1045582
39	3.1733116	1.0960461
36	2.9292107	7.3286800
41	3.3360455	1.4460394
17	1.3832384	9.4218720
17	1.3832384	9.4218720

47	3.8242473	2.9430617
26	2.1155410	2.9616376
14	1.1391375	3.9894873
7	0.5695687	0.0011105
7	0.5695687	0.0011105

Count	%	PValue
84	6.8348250	8.1045582
62	5.0447518	9.1762050
70	5.6956875	8.1060235

Count	%	PValue
20	1.6273393	1.5442547
20	1.6273393	5.6787879
20	1.6273393	2.0945893
20	1.6273393	3.5639020
20	1.6273393	3.5639020
20	1.6273393	6.1305875
20	1.6273393	6.1305875
11	0.8950366	7.6647842
20	1.6273393	7.9702204
11	0.8950366	9.4411086
20	1.6273393	1.3249589
11	0.8950366	1.4196293
11	0.8950366	1.4196293
20	1.6273393	1.6948343
10	0.8136696	2.2492498
20	1.6273393	2.7319164
11	0.8950366	2.9562457
20	1.6273393	3.4436667
21	1.7087062	6.3699101
20	1.6273393	8.3162608
20	1.6273393	1.5431373
21	1.7087062	1.1207663
8	0.6509357	1.1776982
21	1.7087062	1.5262168
21	1.7087062	1.5262168
7	0.5695687	5.1832805
46	3.7428803	1.0874023
21	1.7087062	1.4943022
7	0.5695687	8.0486743
5	0.4068348	0.0010453
25	2.0341741	0.0020469
57	4.6379170	0.0029668
24	1.9528071	0.0043039
23	1.8714401	0.0055621

72	5.8584214	0.0065072
36	2.9292107	0.0074546
30	2.4410089	0.0140543
26	2.1155410	0.0248764
63	5.1261187	0.0456692
24	1.9528071	0.0473336
29	2.3596419	0.0475166

Count	%	PValue
14	1.1391375	3.3234591
14	1.1391375	5.8312106
12	0.9764035	1.1728419
12	0.9764035	1.8406431
10	0.8136696	4.4198212
13	1.0577705	2.9774970
23	1.8714401	0.0025335
22	1.7900732	0.0054016
25	2.0341741	0.0220088
16	1.3018714	0.0234620
23	1.8714401	0.0258092
23	1.8714401	0.0282834

Count	%	PValue
10	0.8136696	4.9465042
25	2.0341741	8.7207000
9	0.7323026	1.5206364
33	2.6851098	2.5163213
9	0.7323026	4.8038702
9	0.7323026	1.3265708
10	0.8136696	2.1658071
35	2.8478437	3.9393400
39	3.1733116	4.9395585
62	5.0447518	9.1762050
20	1.6273393	1.6871967
40	3.2546786	3.1351801
22	1.7900732	3.8592427
23	1.8714401	6.9547685
23	1.8714401	6.9547685
23	1.8714401	6.9547685
41	3.3360455	0.0025434
51	4.1497152	0.0038205
7	0.5695687	0.0102163

Count	%	PValue
10	0.8136696	5.0082115

8	0.6509357	1.2916716
9	0.7323026	1.5510537
8	0.6509357	3.4930076
8	0.6509357	8.6480757
5	0.4068348	0.0017789
17	1.3832384	0.0055049

Count	%	PValue
8	0.6509357	6.2430830
20	1.6273393	6.5056014
8	0.6509357	7.6361075
26	2.1155410	1.9870911
8	0.6509357	2.0828872
17	1.3832384	3.1067505
24	1.9528071	6.7944843
19	1.5459723	1.1269227
5	0.4068348	0.0010453
10	0.8136696	0.0017207
5	0.4068348	0.0024274
31	2.5223759	0.0032096
8	0.6509357	0.0064648
8	0.6509357	0.0103774
8	0.6509357	0.0143083
3	0.2441008	0.0349488
3	0.2441008	0.0349488

Count	%	PValue
20	1.6273393	2.3951210
23	1.8714401	3.8250911
19	1.5459723	1.0553912
19	1.5459723	1.3598527
19	1.5459723	1.5422651
24	1.9528071	1.7322301
20	1.6273393	1.9029479
25	2.0341741	5.9900052
26	2.1155410	6.1665616
25	2.0341741	7.0453541
42	3.4174125	8.7100951
53	4.3124491	0.0107492
44	3.5801464	0.0139627
48	3.9056143	0.0354005

Count	%	PValue
153	12.449145	2.7037436
227	18.470301	0.0441578

162	13.181448	0.0471430

Count	%	PValue
10	0.8136696	4.5502629
8	0.6509357	0.0032518
8	0.6509357	0.0068581

Count	%	PValue
163	13.262815	3.3865876
168	13.669650	4.6292119
165	13.425549	4.9696877
63	5.1261187	0.0384481
120	9.7640358	0.0460527

Count	%	PValue
11	0.8950366	7.5451380
6	0.4882017	2.3081260
5	0.4068348	8.8032724
10	0.8136696	9.9266924
8	0.6509357	0.0011243
5	0.4068348	0.0015326
5	0.4068348	0.0015326
5	0.4068348	0.0017771
5	0.4068348	0.0017771
9	0.7323026	0.0027891
5	0.4068348	0.0028089
12	0.9764035	0.0032730
4	0.3254678	0.0048598
4	0.3254678	0.0072194
4	0.3254678	0.0119965
14	1.1391375	0.0147966
3	0.2441008	0.0218773
3	0.2441008	0.0218773
3	0.2441008	0.0236490
12	0.9764035	0.0252255
6	0.4882017	0.0291602
4	0.3254678	0.0353871

Count	%	PValue
7	0.5695687	0.0014446
9	0.7323026	0.0020489
16	1.3018714	0.0022939
8	0.6509357	0.0027753
7	0.5695687	0.0036636

7	0.5695687	0.0036636
8	0.6509357	0.0041611
8	0.6509357	0.0041611
7	0.5695687	0.0070132
19	1.5459723	0.0091571
3	0.2441008	0.0236127

Count	%	PValue
89	7.2416598	9.2991509
69	5.6143205	5.7138697
52	4.2310821	1.3223490
20	1.6273393	4.1308413
49	3.9869812	5.8607388
31	2.5223759	7.0103118
31	2.5223759	7.0103118
16	1.3018714	0.0022939
102	8.2994304	0.0026603
63	5.1261187	0.0036575
63	5.1261187	0.0039414
23	1.8714401	0.0049002
43	3.4987794	0.0052869
45	3.6615134	0.0056507
18	1.4646053	0.0070928
19	1.5459723	0.0091571
14	1.1391375	0.0096397
21	1.7087062	0.0099410
36	2.9292107	0.0105682
11	0.8950366	0.0109076
11	0.8950366	0.0140875
12	0.9764035	0.0162224
32	2.6037428	0.0286583
10	0.8136696	0.0329187
9	0.7323026	0.0351617
9	0.7323026	0.0351617
15	1.2205044	0.0360202
31	2.5223759	0.0370731
8	0.6509357	0.0395196
14	1.1391375	0.0466898

Count	%	PValue
8	0.6509357	6.6404140
7	0.5695687	0.0023706
7	0.5695687	0.0030314
7	0.5695687	0.0038205
7	0.5695687	0.0047940
10	0.8136696	0.0053321

4	0.3254678	0.0175860
19	1.5459723	0.0277160

Count	%	PValue
21	1.7087062	6.0599696
17	1.3832384	0.0015117
11	0.8950366	0.0017834
15	1.2205044	0.0034919
6	0.4882017	0.0179785
5	0.4068348	0.0384099
5	0.4068348	0.0385224

Count	%	PValue
5	0.4068348	0.0028089
5	0.4068348	0.0036253
5	0.4068348	0.0036253
5	0.4068348	0.0042017
5	0.4068348	0.0050825
6	0.4882017	0.0053507
5	0.4068348	0.0059780
5	0.4068348	0.0059780
5	0.4068348	0.0069796
10	0.8136696	0.0086320
4	0.3254678	0.0108716
4	0.3254678	0.0108716
4	0.3254678	0.0214651
4	0.3254678	0.0214651

Count	%	PValue
6	0.4882017	0.0033887
6	0.4882017	0.0064827
6	0.4882017	0.0064827
6	0.4882017	0.0064827
6	0.4882017	0.0116516
6	0.4882017	0.0118798

Count	%	PValue
9	0.7323026	0.0067336
9	0.7323026	0.0067336
9	0.7323026	0.0067336
9	0.7323026	0.0077902
9	0.7323026	0.0077902

Count	%	PValue
8	0.6509357	7.0641951
18	1.4646053	0.0010979
12	0.9764035	0.0016516
14	1.1391375	0.0025082
13	1.0577705	0.0028814
14	1.1391375	0.0048173
9	0.7323026	0.0049489
9	0.7323026	0.0049489
9	0.7323026	0.0057891
12	0.9764035	0.0066843
9	0.7323026	0.0067336
20	1.6273393	0.0080882
13	1.0577705	0.0085359
3	0.2441008	0.0113105
4	0.3254678	0.0194837
5	0.4068348	0.0202308
22	1.7900732	0.0216581
9	0.7323026	0.0236114
4	0.3254678	0.0345541
9	0.7323026	0.0351617
8	0.6509357	0.0388002
23	1.8714401	0.0497476

Count	%	PValue
4	0.3254678	0.0043275
4	0.3254678	0.0043275
3	0.2441008	0.0490750

Count	%	PValue
4	0.3254678	0.0045523
5	0.4068348	0.0054468
4	0.3254678	0.0376715

Count	%	PValue
7	0.5695687	0.0058083
5	0.4068348	0.0124059
4	0.3254678	0.0396622

Count	%	PValue
13	1.0577705	0.0012697
13	1.0577705	0.0248527
14	1.1391375	0.0322224
14	1.1391375	0.0419627

Count	%	PValue
8	0.6509357	0.0074260
8	0.6509357	0.0163041
8	0.6509357	0.0360008

Count	%	PValue
11	0.8950366	0.0067726
11	0.8950366	0.0069062
10	0.8136696	0.0070277
23	1.8714401	0.0134109
12	0.9764035	0.0136552
8	0.6509357	0.0173330
11	0.8950366	0.0187638
12	0.9764035	0.0267767
5	0.4068348	0.0350344
8	0.6509357	0.0360008
11	0.8950366	0.0480658

Count	%	PValue
6	0.4882017	0.0124658
7	0.5695687	0.0177319
16	1.3018714	0.0234620

Count	%	PValue
7	0.5695687	0.0164440
7	0.5695687	0.0190128
6	0.4882017	0.0213760

Count	%	PValue
12	0.9764035	0.0071095
5	0.4068348	0.0346188
5	0.4068348	0.0346188

Count	%	PValue
4	0.3254678	0.0119965
5	0.4068348	0.0236265
5	0.4068348	0.0451932

Count	%	PValue
4	0.3254678	0.0080911

4	0.3254678	0.0155544
8	0.6509357	0.0323583
3	0.2441008	0.0377119
3	0.2441008	0.0490750

Count	%	PValue
22	1.7900732	0.0111100
25	2.0341741	0.0247820
15	1.2205044	0.0382729
12	0.9764035	0.0481730

Count	%	PValue
11	0.8950366	0.0217048
6	0.4882017	0.0290502
4	0.3254678	0.0396622

Genes	List Tota
KRT6C, KRT6A, KRT6B, KRT23, KRT33B, KRT81, KRT9, KRT25, KRT	1206
KRT6C, KRT6A, KRT6B, LMNB2, KRT23, KRT33B, KRT81, KRT9, KRT	1206
KRT6C, KRT6A, KRT6B, LMNB2, KRT23, KRT33B, KRT81, KRT9, KRT	1206
KRT6C, KRT6A, KRT6B, LMNB2, KRT23, KRT33B, KRT81, KRT9, KRT	1206
KRT6C, KRT6A, KRT6B, LMNB2, KRT23, KRT33B, KRT81, KRT9, KRT	1206
KRT6C, KRT6A, KRT6B, LMNB2, KRT23, KRT33B, KRT81, KRT9, KRT	1206
KRT6C, KRT6A, KRT6B, LMNB2, KRT23, KRT33B, KRT81, KRT9, KRT	1206
KRT6C, KRT6A, KRT6B, LMNB2, KRT23, KRT33B, KRT81, KRT9, KRT	1208
KRT6C, KRT6A, KRT6B, LMNB2, KRT33B, KRT81, KRT9, KRT25, KRT	1206
KRT6C, KRT6A, LMNB2, KRT23, KRT33B, KRT81, KRT9, KRT25, KRT	1095
KRT6C, KRT6A, LMNB2, KRT23, KRT33B, KRT81, KRT9, KRT25, KRT	1095
KRT6C, KRT6A, KRT6B, LMNB2, KRT23, KRT81, KRT9, KRT25, KRT2	561
KRT6C, KRT6A, LMNB2, KRT23, KRT33B, KRT81, KRT9, KRT25, KRT	1095
KRT6C, KRT6A, KRT81, KRT72, KRT75, KRT74, KRT80, KRT77, KRT	1095
LOR, RPL36A, TUBB2A, RPL22L1, KRT33B, KRT81, KRT80, RPL39L,	853
KRT6C, KRT6A, KRT6B, KRT31, KRT34, KRT33B, KRT72, KRT75, KR	1206
KRT6C, KRT6A, KRT6B, KRT23, KRT33B, KRT81, KRT9, KRT25, KRT	1208
KRT6C, KRT6A, KRT6B, LMNB2, KRT23, KRT33B, KRT81, KRT9, KRT	908
KRT6C, KRT6A, KRT6B, KRT10, KRT81, KRT9, KRT72, KRT75, KRT7	908
KRT6C, KRT6A, KRT6B, LMNB2, KRT23, KRT33B, KRT81, KRT9, KRT	908
KRT9, KRT25, KRT28, KRT17, KRT16, KRT15, KRT14, KRT31, KRT1	1095

Genes	List Tota
LOR, PPARD, LCE3A, PTGS2, S100A7, SPINK5, PLOD1, LCE2D, KRT	924
LOR, PPARD, PTGS2, LCE3A, S100A7, SPINK5, PLOD1, LCE2D, KRT	924
LOR, LCE4A, LCE3A, LCE5A, LCE6A, LCE2C, LCE2B, LCE2A, SPRR2	1208
LOR, LCE4A, LCE3A, LCE5A, LCE6A, LCE2C, LCE2B, LCE2A, SPRR2	924
LOR, LCE3A, S100A7, LCE5A, LCE1B, SPRR2F, SPRR2E, LCE1A, SP	924
LOR, LCE3A, S100A7, LCE5A, LCE1B, SPRR2F, SPRR2E, LCE1A, SP	924
LCE5A, LCE2C, LCE2B, SPRR2F, LCE1B, LCE2A, SPRR2E, LCE1A, L	1095
LOR, LCE3A, S100A7, LCE5A, LCE1B, SPRR2F, SPRR2E, LCE1A, SP	924
LOR, LCE3A, S100A7, LCE5A, HOXA11, LCE1B, SPRR2F, SPRR2E, L	924
LOR, SPRR2D, SPRR1A, RPTN, SPRR1B, SPRR2A, TGM1, SPRR2F, SP	908

Genes	List Tota
RALY, RPL36A, SNRPD3, LSM6, SNRPD1, RPL22L1, PCBP4, PCBP1,	1208
RALY, RPP38, RPL36A, SNRPD3, LSM6, SNRPD1, RPS6KB2, RPL22L1	908
MRPS17, RPL36A, MRPS15, MRPS12, RPS6KB2, RPL22L1, RPL39, MR	1208
MRPS17, RPL36A, MRPS15, MRPS12, RPL22L1, RPL39, MRPL11, RPL	853
MRPS17, RPL36A, MRPS15, MRPS12, RPS6KB2, RPL22L1, RPL39, MR	908
MRPL1, MRPS17, MRPL51, MRPS15, MRPS22, MRPS12, MRPL9, MRPS2	908
MRPL1, MRPS17, MRPL51, MRPS15, MRPS22, MRPS12, MRPL9, MRPS2	908

MRPS17, RPL36A, MRPS15, PSTK, MRPS12, PABPC4, RPS6KB2, RPL2	924
MRPS17, MRPS15, MRPS12, RPL39, MRPL11, RPS25, RPL30, RPL7,	908
MRPS17, MRPS15, MRPS22, MRPS12, MRPS21, MRPS6, RPS7, RPS25,	908
MRPS17, MRPS18C, MRPS15, MRPS9, MRPS18A, MRPS22, MRPS21	908
MRPS17, MRPS18C, MRPS15, MRPS9, MRPS18A, MRPS22, MRPS21	908

Genes	List Tota
RALY, RPP38, RPL36A, SNRPD3, LSM6, SNRPD1, RPS6KB2, RPL22L1	908
RPP38, RALY, RPL36A, LSM6, SNRPD3, SNRPD1, RP9, YBX1, PCBP1	924
RALY, RPL36A, LSM6, SNRPD1, TIMM50, YBX1, PCBP4, PCBP1, U2A	853

Genes	List Tota
SHFM1, PSMA7, PSMB5, PSMA2, PSMB4, PSMB7, PSMB1, PSMC4, PSM	325
SHFM1, PSMA7, PSMB5, PSMA2, PSMB4, PSMB7, PSMB1, PSMC4, PSM	1208
SHFM1, PSMA7, PSMB5, PSMA2, PSMB4, PSMB7, PSMB1, PSMC4, PSM	908
UBE2C, PSMA7, PSMB5, PSMA2, PSMB4, PSMB7, PSMB1, PSMC4, PSM	924
UBE2C, PSMA7, PSMB5, PSMA2, PSMB4, PSMB7, PSMB1, PSMC4, PSM	924
UBE2C, PSMA7, PSMB5, PSMA2, PSMB4, PSMB7, PSMB1, PSMC4, PSM	924
UBE2C, PSMA7, PSMB5, PSMA2, PSMB4, PSMB7, PSMB1, PSMC4, PSM	924
PSMA2, PSMB5, PSMB4, PSMB7, PSMB1, PSMA5, PSMA4, PSMB3, PSM	1095
UBE2C, PSMA7, PSMB5, PSMA2, PSMB4, PSMB7, PSMB1, PSMC4, PSM	924
PSMA2, PSMB5, PSMB4, PSMB7, PSMB1, PSMA5, PSMA4, PSMB3, PSM	1208
UBE2C, PSMA7, PSMB5, PSMA2, PSMB4, PSMB7, PSMB1, PSMC4, PSM	924
PSMA2, PSMB5, PSMB4, PSMB7, PSMB1, PSMA5, PSMA4, PSMB3, PSM	853
PSMA2, PSMB5, PSMB4, PSMB7, PSMB1, PSMA5, PSMA4, PSMB3, PSM	853
UBE2C, PSMA7, PSMB5, PSMA2, PSMB4, PSMB7, PSMB1, PSMC4, PSM	924
PSMA2, PSMB5, PSMB4, PSMB1, PSMA5, PSMA4, PSMB3, PSMA3, PSM	1208
UBE2C, PSMA7, PSMB5, PSMA2, PSMB4, PSMB7, PSMB1, PSMC4, PSM	924
PSMA2, PSMB5, PSMB4, PSMB7, PSMB1, PSMA5, PSMA4, PSMB3, PSM	908
UBE2C, PSMA7, PSMB5, PSMA2, PSMB4, PSMB7, PSMB1, PSMC4, PSM	924
NHLRC1, UBE2C, PSMA7, PSMB5, PSMA2, PSMB4, PSMB7, PSMB1, PS	924
UBE2C, PSMA7, PSMB5, PSMA2, PSMB4, PSMB7, PSMB1, PSMC4, PSM	924
UBE2C, PSMA7, PSMB5, PSMA2, PSMB4, PSMB7, PSMB1, PSMC4, PSM	924
NHLRC1, UBE2C, PSMA7, PSMB5, PSMA2, PSMB4, PSMB7, PSMB1, PS	924
PSMB5, PSMB4, PSMB7, PSMB1, PSMA5, PSMB3, PSMB2, PSMA7	1095
UBE2G2, UBE2C, PSMA7, PSMB5, PSMA2, PSMB4, PSMB7, PSMB1, PS	924
UBE2G2, UBE2C, PSMA7, PSMB5, PSMA2, PSMB4, PSMB7, PSMB1, PS	924
PSMB5, PSMB4, PSMB7, PSMB1, PSMA4, PSMB3, PSMB2	1095
TUBB2A, KATNB1, PKMYT1, PTTG2, PTTG1, RCC1, PSMA7, PSMB5, P	924
NF2, UBE2C, PSMA7, PSMB5, PSMA2, PSMB4, PSMB7, PSMB1, PSMC4	924
PSMA2, PSMB1, PSMA4, PSMA3, PSMD7, PSMD8, RPS27A	1208
PSMA2, PSMA5, PSMA4, PSMA3, PSMA7	1095
IGF2BP1, PSMA7, PSMB5, PSMB4, PSMB7, PSMB1, PSMB3, PSMB2, P	924
TUBB2A, PKMYT1, PTTG2, PTTG1, STRA8, PCBP4, PSMD2, PSMD4, P	924
IGF2, NHLRC1, IL34, UBE2C, PSMA7, IL11, PSMB5, PSMA2, PSMB4	924
NF2, IGF2BP1, IGF2, UBE2C, PSMA7, PSMB5, PSMA2, PSMB4, PSME	924

STEAP3, S100A6, TUBB2A, PKMYT1, PTTG2, PTTG1, STRA8, PCBP4,	924
HR, SFN, PSMA7, PSMB5, PSMB4, PSMB7, PSMB1, WWP2, PSMB3, PS	924
SFN, PSMA7, PSMB5, PSMB4, PSMB7, PSMB1, PSMB3, PSMB2, PSMD2	924
USP5, UCHL1, UBE2G2, PSMA7, PSMB5, PSMB4, PSMB7, PSMB1, PSM	924
PPARD, HR, MAF1, NR2E1, KCNIP3, TBPL2, SAP30, STRA8, WWP2,	924
IGF2, NHLRC1, IL34, UBE2C, PSMA7, IL11, PSMB5, PSMA2, PSMB4	924
NHLRC1, IL34, PSMA7, IL11, PSMB5, MAP3K6, PSMB4, PSMB7, PSM	924

Genes	List Tota
S100A4, S100A6, S100P, S100A16, S100A8, S100A7, S100A9, S10	1095
S100A4, S100A6, S100P, S100A16, S100A8, S100A7, S100A9, S10	1095
S100A4, S100A16, S100P, S100A8, S100A7, RPTN, S100A9, S100A	1206
S100A4, S100A6, S100P, S100A8, S100A7, S100A9, S100A11, S10	561
S100A4, S100A16, S100P, S100A8, RPTN, S100A9, S100A11, S100	1206
S100A4, S100A6, S100P, HPCAL1, S100A8, S100A7, S100A9, S100	1208
S100A4, S100A6, S100A16, S100P, HPCAL1, S100A8, NCALD, TNNC	1206
S100A4, S100A6, S100A16, HPCAL1, S100P, S100A8, NCALD, TNNC	1206
S100A4, S100A6, S100A8, TNNC1, S100A7, S100A9, NCS1, NECAB2	1095
S100A6, HPCAL1, S100A8, TNNC1, NCALD, S100A9, S100A11, NCS1	1095
S100A4, S100A6, S100A16, S100P, HPCAL1, S100A8, NCALD, TNNC	1095
S100A4, S100A6, S100A16, S100P, HPCAL1, S100A8, NCALD, TNNC	1095

Genes	List Tota
SNRPA1, SNRPD3, SNRPB2, U2AF1, SNRPD1, SNRPA, SNRPC, SNRPF,	94
SNRPA1, NHP2L1, MAGOH, SNRPD3, LSM6, SNRPB2, SNRPD1, SF3B4,	325
LSM6, SNRPD3, SNRPD1, LSM5, LSM3, LSM2, SNRPF, SNRPE, SNRPG	514
RALY, NHP2L1, LSM6, SNRPD3, SNRPB2, SNRPD1, SF3B4, YBX1, SF	1208
LSM6, SNRPD3, SNRPD1, LSM5, LSM3, LSM2, SNRPF, SNRPE, SNRPG	1095
LSM6, SNRPD3, SNRPD1, LSM5, LSM3, LSM2, SNRPF, SNRPE, SNRPG	1095
SNRPA1, LSM6, SNRPD3, SNRPB2, SNRPD1, PHF5A, SNRPC, SNRPF,	908
RALY, NHP2L1, LSM6, SNRPD3, SNRPB2, SNRPD1, SF3B4, YBX1, SF	1208
POLR2H, RALY, RPL36A, POLR2F, NHP2L1, LSM6, SNRPD3, SNRPB2,	924
RPP38, RALY, RPL36A, LSM6, SNRPD3, SNRPD1, RP9, YBX1, PCBP1	924
RALY, SNRPA1, NHP2L1, MAGOH, SNRPD3, SNRPB2, SNRPD1, SF3B4,	1208
POLR2H, RALY, ZC3H3, POLR2F, RPL36A, NHP2L1, LSM6, SNRPD3,	924
RALY, TXNL4B, SNRPA1, NHP2L1, MAGOH, SNRPD3, SNRPB2, SNRPD1	908
POLR2H, SNRPA1, RPL36A, POLR2F, NHP2L1, MAGOH, SNRPD3, PTBF	924
POLR2H, SNRPA1, RPL36A, POLR2F, NHP2L1, MAGOH, SNRPD3, PTBF	924
POLR2H, SNRPA1, RPL36A, POLR2F, NHP2L1, MAGOH, SNRPD3, PTBF	924
POLR2H, RALY, ZC3H3, POLR2F, RPL36A, NHP2L1, LSM6, SNRPD3,	924
RALY, RPL36A, LSM6, SNRPD3, TIMM50, YBX1, PCBP4, PCBP1, LSM	1208
SNRPD3, WDR77, SNRPD1, SNRPC, SNRPF, SNRPE, SNRPG	924

Genes	List Tota
LYPD2, LYPD1, SLURP1, LYNX1, LYPD3, LY6D, LY6E, LYPD5, LY6G	1095

LYPD2, LYPD1, SLURP1, LYNX1, LYPD3, LY6D, LY6E, PLAUR	514
LYPD2, LYPD1, SLURP1, LYNX1, LY6D, LY6E, LYPD5, LY6G6C, LY6	1206
LYPD2, LYPD1, SLURP1, LYNX1, LYPD3, LY6D, LY6E, PLAUR	1095
LYPD2, SLURP1, LYNX1, LYPD3, LY6D, LY6E, LYPD5, PLAUR	1095
LYPD2, LYNX1, LY6D, LY6E, LY6K	561
LYPD2, LYPD1, LYPD3, LYPD5, MFI2, PRSS41, LY6G6C, RTN4R, LY	1208

Genes	List Tota
WFDC12, WFIKKN2, UMODL1, PI3, SLPI, WFDC1, WFDC5, WFDC3	1095
WFIKKN2, LRIT2, SERPINA12, SPINT1, SERPINH1, SPINK5, SPINK7	853
WFDC12, WFIKKN2, UMODL1, PI3, SLPI, WFDC1, WFDC5, WFDC3	514
WFIKKN2, LRIT2, SERPINA12, PTTG2, PTTG1, SERPINH1, SPINK5,	853
WFDC12, WFIKKN2, UMODL1, PI3, SLPI, WFDC1, WFDC5, WFDC3	1095
WFIKKN2, SERPINA12, SPINT1, SPINK5, SPINK7, WFDC12, PI3, SE	1208
WFIKKN2, LRIT2, SERPINA12, PTTG2, PTTG1, SERPINH1, SPINK5,	853
KNG1, WFIKKN2, SERPINA12, SPINT1, R3HDML, SPINK5, SPINK7, W	1208
WFDC12, PI3, SLPI, WFDC5, WFDC3	1095
WFIKKN2, SERPINB2, SPINT1, SERPINB11, SERPINB4, SERPINB12,	1206
WFDC12, WFIKKN2, UMODL1, PI3, WFDC1	1206
WFIKKN2, LRIT2, SERPINA12, RTKN, PTTG2, SFN, PTTG1, SERPINH	853
SERPINA12, SERPINB2, SERPINB11, SERPINB4, SERPINB12, SERPIN	514
SERPINA12, SERPINB2, SERPINB11, SERPINB4, SERPINB12, SERPIN	561
SERPINA12, SERPINB2, SERPINB11, SERPINB4, SERPINB12, SERPIN	1095
SLPI, WFDC5, WFDC3	1206
SLPI, WFDC5, WFDC3	1206

Genes	List Tota
KLK6, KLK7, KLK8, KLK9, PRSS41, KLK5, KLK1, TMPRSS5, HTRA2,	514
KLK6, KLK7, KLK8, KLK9, PRSS41, KLK5, KLK1, TMPRSS5, PARL,	1208
KLK6, KLK7, KLK8, KLK9, PRSS41, KLK5, KLK1, TMPRSS5, TMPRSS	1206
KLK6, KLK7, KLK8, KLK9, PRSS41, KLK5, KLK1, TMPRSS5, TMPRSS	1095
KLK6, KLK7, KLK8, KLK9, PRSS41, KLK5, KLK1, TMPRSS5, TMPRSS	1095
KLK6, KLK7, KLK8, KLK9, PRSS41, KLK5, KLK1, TMPRSS5, PWP2,	853
KLK6, KLK7, KLK8, KLK9, PRSS41, KLK5, KLK1, TMPRSS5, HTRA2,	1095
PRSS41, HTRA2, PARL, PRSS3, PCSK9, KLK10, PRSS33, KLK15, TM	853
PRSS41, NOTUM, HTRA2, PRSS3, PCSK9, KLK10, PRSS33, PTS, KLK	1206
PRSS41, HTRA2, PARL, PRSS3, PCSK9, KLK10, PRSS33, KLK15, TM	853
PDIA3, USP5, PRSS41, UCHL1, RCE1, PSMA7, PSMB5, PSMB4, PSMB	853
PDIA3, UCHL1, PRSS41, RCE1, SPINK5, PARL, HTRA2, KLK10, PRS	853
USP5, PRSS41, UCHL1, PSMA7, SPINK5, PSMB5, PSMB4, PSMB7, HT	1208
PDIA3, USP5, PRSS41, UCHL1, RCE1, PSMA7, PSMB5, PSMB4, PSMB	853

Genes	List Tota
PVR, S100A8, S100A7, S100A9, IL17RE, NOTUM, IL11, REG3A, PG	1208
PVR, LYPD2, LYPD1, LYPD3, PTGS2, LYPD5, L1CAM, NOTUM, PLOD1	1208

PVR, S100A7, IL17RE, NOTUM, IL11, REG3A, PGLYRP4, ST3GAL4,	908

Genes	List Tota
MAGED1, TYR, MAGEA12, TACSTD2, MAGEA2, MAGEA11, MAGEA6, MAG	1208
MAGED1, MAGEA12, MAGEA2, MAGEA11, MAGEA6, MAGED4, MAGEA3, M	1206
MAGED1, MAGEA12, MAGEA2, MAGEA11, MAGEA6, MAGED4, MAGEA3, M	1095

Genes	List Tota
S100A4, RPP38, LOR, RPL36A, PTGS2, SNRPD3, XRCC6, S100A9, H	908
S100A4, RPP38, LOR, RPL36A, PTGS2, SNRPD3, XRCC6, S100A9, H	908
S100A4, RPP38, LOR, RPL36A, PTGS2, SNRPD3, XRCC6, S100A9, H	908
RPP38, S100A4, RPL36A, LYAR, SNRPD3, UCHL1, S100A9, TCOF1,	908
S100A4, RPP38, LOR, RPL36A, SNRPD3, S100A9, XRCC6, HOXD12,	908

Genes	List Tota
P4HB, TXNDC12, PDIA3, NXN, PRDX6, TXN, PRDX4, PDIA6, PDIA5,	1095
P4HB, PDIA3, TXN, PDIA6, PDIA5, PDIA4	1095
P4HB, PDIA3, TXN, PDIA6, PDIA4	1208
P4HB, TXNDC12, PDIA3, PRDX6, TXN, PRDX4, PDIA6, PDIA5, PDIA	1208
P4HB, TXNDC12, PDIA3, NXN, TXN, PDIA6, PDIA5, PDIA4	1095
P4HB, PDIA3, PDIA6, PDIA5, PDIA4	1206
P4HB, PDIA3, PDIA6, PDIA5, PDIA4	1206
P4HB, PDIA3, PDIA6, PDIA5, PDIA4	853
P4HB, PDIA3, PDIA6, PDIA5, PDIA4	853
P4HB, TPI1, PDIA3, PDIA6, PDIA5, RPIA, IDI2, PDIA4, SIGMAR1	853
P4HB, PDIA3, PDIA6, PDIA5, PDIA4	853
P4HB, TXNDC12, PDIA3, NXN, PRDX6, TXN, PRDX4, PDIA6, PDIA5,	924
P4HB, PDIA3, PDIA6, PDIA4	1095
P4HB, PDIA3, TXN, PDIA6	1206
P4HB, PDIA3, PDIA6, PDIA5	561
TXNL4B, P4HB, PDIA3, PRDX4, PDIA6, PDIA5, PDIA4, PRDX1, TXN	1095
P4HB, PDIA3, PDIA6	1206
P4HB, PDIA3, PDIA6	1206
P4HB, PDIA3, PDIA4	1095
P4HB, TXNDC12, PDIA3, PTGS2, P4HA2, PDIA6, SUMF2, IGF2, PDI	908
P4HB, PDIA3, TXN, PDIA6, PDIA5, PDIA4	1095
P4HB, TPI1, PDIA3, PDIA4	1208

Genes	List Tota
SDHA, SDHB, SUCLG1, SDHC, IDH2, MDH2, MDH1	1208
SDHA, SDHB, UQCRH, SUCLG1, SDHC, IDH2, MDH2, UQCRB, MDH1	924
NDUFB3, NDUFA4, NDUFB5, SUCLG1, NDUFA6, NDUFB9, SDHA, SDHB,	924
SDHA, SDHB, SUCLG1, SDHC, IDH2, PDHA1, MDH2, MDH1	325
SDHA, SDHB, SUCLG1, SDHC, IDH2, MDH2, MDH1	924

SDHA, SDHB, SUCLG1, SDHC, IDH2, MDH2, MDH1	924
SDHA, SDHB, TDO2, SUCLG1, SDHC, IDH2, MDH2, MDH1	924
SDHA, SDHB, UGT1A8, SUCLG1, SDHC, IDH2, MDH2, MDH1	924
SDHA, SDHB, SUCLG1, SDHC, IDH2, MDH2, MDH1	924
NDUFB3, PRKAG3, NDUFA4, NDUFB5, NDUFB9, SUCLG1, NDUFA6, SDH	924
SDHA, SDHB, SDHC	853

Genes	List Tota
SMCP, TIMM50, UQCRFS1, MFF, NDUFS5, HTRA2, PARL, MRPL36, SI	1208
STOML2, TIMM50, UQCRFS1, MFF, NDUFS5, HTRA2, PARL, MRPL36,	908
TIMM50, UQCRFS1, PARL, HTRA2, SLC25A3, MRPL36, MRPL37, PDHA	1208
NDUFB3, NDUFA4, NDUFB5, SLC25A5, NDUFA4L2, NDUFB9, NDUFA6,	325
TIMM50, UQCRFS1, PARL, HTRA2, SLC25A3, MRPL36, MRPL37, PDHA	1206
MRPS17, MRPS15, MRPS12, KARS, MRPL11, MRPL16, MRPL36, MRPL3	908
MRPS17, MRPS15, MRPS12, KARS, MRPL11, MRPL16, MRPL36, MRPL3	908
NDUFB3, NDUFA4, NDUFB5, SUCLG1, NDUFA6, NDUFB9, SDHA, SDHB,	924
NIT2, STOML2, MFF, HTRA2, MRPL36, MRPL37, PDHA1, MRPL33, TM	908
S100A6, HTATIP2, LEMD2, STOML2, TIMM50, RANGAP1, UQCRFS1, M	908
S100A6, HTATIP2, LEMD2, STOML2, TIMM50, RANGAP1, UQCRFS1, M	908
NDUFB3, NDUFA4, NDUFB5, SLC25A5, NDUFB9, NDUFA6, TIMM50, UQ	1208
NDUFB3, NDUFB5, SAMM50, NDUFB9, STOML2, TIMM50, UQCRFS1, MF	908
NDUFB3, NDUFB5, SAMM50, NDUFB9, STOML2, TIMM50, UQCRFS1, MF	908
NDUFB3, NDUFA4, NDUFB5, SAMM50, NDUFB9, NDUFA6, TOMM40, TIM	908
NDUFB3, PRKAG3, NDUFA4, NDUFB5, NDUFB9, SUCLG1, NDUFA6, SDH	924
SDHA, NDUFB3, NDUFA4, SDHB, NDUFS5, NDUFB5, UQCRH, NDUFB9,	1208
NDUFB3, POLR2H, NDUFA4, POLR2F, NDUFB5, SLC25A5, NDUFA4L2,	325
NDUFB3, NDUFB5, LMNB2, NDUFB9, LEMD2, STOML2, TIMM50, UQCRF	908
SDHA, NDUFB3, NDUFA4, SDHB, NDUFS5, NDUFB5, UQCRH, NDUFB9,	924
SDHA, NDUFB3, NDUFA4, NDUFS5, NDUFB5, UQCRH, NDUFB9, NDUFA6	908
SDHA, NDUFB3, NDUFA4, NDUFS5, NDUFB5, UQCRH, NDUFB9, NDUFA6	908
NDUFB3, NDUFB5, NDUFB9, STOML2, TIMM50, UQCRFS1, NDUFS5, PA	908
NDUFB3, NDUFA4, NDUFS5, NDUFB5, UQCRH, NDUFB9, NDUFA6, UQCF	1208
NDUFB3, NDUFA4, NDUFS5, NDUFB5, UQCRH, NDUFB9, NDUFA6, NDUF	924
NDUFB3, NDUFA4, NDUFS5, NDUFB5, UQCRH, NDUFB9, NDUFA6, NDUF	924
NDUFA4, NDUFB3, NDUFB5, NDUFA4L2, NDUFA6, NDUFB9, UQCRFS1,	325
NDUFB3, PRKAG3, NDUFB5, PPARD, NDUFB9, PGAM1, POMC, UQCRFS1	924
NDUFB3, NDUFA4, NDUFS5, NDUFB5, TP53I3, NDUFB9, NDUFA6, NDU	853
SDHA, NDUFB3, NDUFA4, SDHB, NDUFS5, NDUFB5, UQCRH, NDUFB9,	924

Genes	List Tota
TUBA8, MSTO1, TUBB2A, TUBB6, TUBA4A, TUBA1B, TUBA1C, TUBB3	1095
TUBA8, TUBB2A, TUBB6, TUBA4A, TUBA1B, TUBA1C, TUBB3	1095
TUBA8, TUBB2A, TUBB6, TUBA4A, TUBA1B, TUBA1C, TUBB3	1095
TUBA8, TUBB2A, TUBB6, TUBA4A, TUBA1B, TUBA1C, TUBB3	1095
TUBA8, TUBB2A, TUBB6, TUBA4A, TUBA1B, TUBA1C, TUBB3	561
TUBBP5, TUBA8, MSTO1, TUBB2A, RAC1, TUBB6, TUBA4A, TUBA1B,	924

TUBA8, TUBA4A, TUBA1B, TUBA1C	1095
MSTO1, OOEP, SAMM50, IPO13, TUBB2A, THEG, SRC, TUBBP5, PSM	924

Genes	List Tota
SLC16A13, SLC36A2, SLC6A10P, SLC15A1, SLC34A1, SLC6A16, SL	853
SLC16A13, SLC6A10P, SLC15A1, SLC34A1, SLC6A16, SLC6A18, SL	1208
SLC1A5, SLC6A9, SLC6A10P, SLC6A8, SLC1A6, SLC5A9, SLC6A16,	853
SLC36A2, SLC6A10P, SLC15A1, SLC6A16, SLC6A18, SLC10A3, SLC	853
SLC6A9, SLC6A10P, SLC6A8, SLC18A3, SLC6A16, SLC6A18	853
SLC6A9, SLC6A10P, SLC6A8, SLC6A16, SLC6A18	853
SLC6A9, SLC6A10P, SLC6A8, SLC6A16, SLC6A18	1095

Genes	List Tota
MT1L, NOS1, MT1E, MT1G, MT1X, MT1F	853
MT1L, MT1E, MT4, MT1G, MT1X, MT1F	1206
MT1L, MT1E, MT4, MT1G, MT1X, MT1F	1206
MT1L, MT1E, MT4, MT1G, MT1X, MT1F	1095
MT1L, MT1E, MT4, MT1G, MT1X, MT1F	1208
MT1L, ZNF133, MFI2, MT1E, MT1G, MT1X, MT1F	1208
MT1L, MT1E, MT4, MT1G, MT1X, MT1F	1095
MT1L, MT1E, MT4, MT1G, MT1X, MT1F	1095
MT1L, MT1E, MT4, MT1G, MT1X, MT1F	561
STEAP3, TYRP1, TYR, MT1L, MT1E, MT4, MT1G, SOD3, MT1X, S10	1208
MT1L, MT1E, MT1G, MT1X, MT1F	1208
MT1L, MT1E, MT1G, MT1X, MT1F	1208
MT1L, MT1E, MT1G, MT1X, MT1F	1206
MT1L, MT1E, MT1G, MT1X, MT1F	1206

Genes	List Tota
WNT10A, WNT10B, WNT5B, WNT11, WNT6, WNT7A	514
WNT10A, WNT10B, WNT5B, WNT11, WNT6, WNT7A	1095
WNT10A, WNT10B, WNT5B, WNT11, WNT6, WNT7A	1095
WNT10A, WNT10B, WNT5B, WNT11, WNT6, WNT7A	1095
WNT10A, WNT10B, WNT5B, WNT11, WNT6, WNT7A	561
WNT10A, WNT10B, WNT5B, WNT11, WNT6, WNT7A	924

Genes	List Tota
KRT25, PPARD, PTGS2, HOXC13, RELA, SNAI1, SPINK5, KRT71, N	924
KRT25, PPARD, PTGS2, HOXC13, RELA, SNAI1, SPINK5, KRT71, N	924
KRT25, PPARD, PTGS2, HOXC13, RELA, SNAI1, SPINK5, KRT71, N	924
KRT25, PPARD, PTGS2, HOXC13, RELA, SNAI1, SPINK5, KRT71, N	924
KRT25, PPARD, PTGS2, HOXC13, RELA, SNAI1, SPINK5, KRT71, N	924

Genes	List Tota
TALDO1, PFKL, PGM1, PGD, PFKP, TKT, RPIA, TKTL1	325
TALDO1, PFKL, PGD, NUDT5, PGAM1, PFKP, TKTL1, HYAL4, TPI1,	924
TPI1, TALDO1, PFKL, PGM1, PGD, PGAM1, PFKP, PDHA1, RPIA, TK	924
TYRP1, TALDO1, RBP1, PGD, PNP, RDH12, TPI1, TP53I3, TYR, TD	924
TALDO1, PFKL, NUDT5, PGD, PGAM1, PFKP, TKTL1, TPI1, PGM1, F	924
TALDO1, PFKL, NUDT5, PGD, PGAM1, PFKP, TKTL1, TPI1, PGM1, F	924
TDO2, TPI1, TP53I3, TALDO1, PGD, RPIA, PNP, MDH2, MDH1	924
TDO2, TPI1, TP53I3, TALDO1, PGD, RPIA, PNP, MDH2, MDH1	924
TDO2, TPI1, TP53I3, TALDO1, PGD, RPIA, PNP, MDH2, MDH1	924
TPI1, TALDO1, PFKL, PGM1, PGD, PGAM1, PFKP, PDHA1, RPIA, TK	924
TDO2, TPI1, TP53I3, TALDO1, PGD, RPIA, PNP, MDH2, MDH1	924
PRKAG3, PPARD, TALDO1, PFKL, SERPINA12, SLC37A4, PGD, PGAM1	924
TALDO1, PFKL, NUDT5, PGD, PGAM1, PFKP, TKTL1, TPI1, PGM1, F	924
TPI1, TALDO1, PGD	1208
TPI1, TALDO1, PGD, RPIA	924
TPI1, TP53I3, TALDO1, PGD, RPIA	924
PRKAG3, PPARD, TALDO1, PFKL, SERPINA12, SLC37A4, PGD, PGAM1	924
TDO2, TPI1, TP53I3, TALDO1, PGD, RPIA, PNP, MDH2, MDH1	924
TALDO1, NUDT5, PGD, RPIA	924
TDO2, TPI1, TP53I3, TALDO1, PGD, RPIA, PNP, MDH2, MDH1	924
TPI1, PFKL, PGM1, PGAM1, PFKP, PDHA1, MDH2, MDH1	924
PRKAG3, PPARD, TALDO1, PFKL, SERPINA12, SLC37A4, PGD, NUDT5	924

Genes	List Tota
RAET1E, RAET1G, ULBP2, RAET1L	1206
RAET1E, RAET1G, ULBP2, RAET1L	1206
RAET1E, ULBP2, RAET1L	561

Genes	List Tota
RANBP1, RANGAP1, SNHG3, RCC1	94
NUP62, NUTF2, RANGAP1, SNHG3, RCC1	94
RANBP1, RANGAP1, SNHG3, RCC1	94

Genes	List Tota
RPUSD4, PUS3, PUS1, RPUSD3, PGM1, PGAM1, LSS	853
RPUSD4, PUS3, PUS1, RPUSD3, NOP10	924
RPUSD4, PUS3, PUS1, RPUSD3	853

Genes	List Tota
MSTO1, SAMM50, TOMM40, PI4KB, VDAC2, MFF, TOMM6, TOMM5, TOM	1208
MSTO1, SAMM50, TOMM40, PI4KB, VDAC2, MFF, TOMM6, TOMM5, TOM	908
MSTO1, SAMM50, TOMM40, PI4KB, VDAC2, SIGMAR1, MFF, TOMM6, T	908
MSTO1, SAMM50, TOMM40, PI4KB, VDAC2, SIGMAR1, MFF, TOMM6, T	908

Genes	List Tota
UBE2G2, UBE2I, TMEM189, UBE2L3, UBE2C, UBE2S, UBE2Q1, UBE2E	514
UBE2G2, UBE2I, TMEM189, UBE2L3, UBE2C, UBE2S, UBE2Q1, UBE2E	1095
UBE2G2, UBE2I, TMEM189, UBE2L3, UBE2C, UBE2S, UBE2Q1, UBE2E	1095

Genes	List Tota
TRIM16L, TRIM65, TRIM47, TRIM48, TRIM43, TRIM50, TRIM16, RF	1095
TRIM55, TRIM65, TRIM47, TRIM48, TRIM43, TRIM29, TRIM31, TRI	514
TRIM55, TRIM65, TRIM47, TRIM48, TRIM43, TRIM29, TRIM31, TRI	1206
RNF17, RNF220, TRIM50, NHLRC1, TRIM15, TRIM10, TRAIP, RNF22	1206
TRIM16L, TRIM65, TRIM47, TRIM48, HNRNPUL2, TRIM43, TRIM50,	1206
TRIM16L, TRIM47, TRIM50, TRIM16, RFPL3, TRIM15, TRIM10, TRI	514
TRIM55, TRIM65, TRIM47, TRIM48, TRIM43, TRIM29, TRIM31, TRI	1095
TRIM16L, TRIM65, TRIM47, TRIM48, HNRNPUL2, TRIM43, TRIM50,	1095
TRIM43, TRIM50, TRIM15, TRIM10, TRIML1	561
TRIM16L, TRIM47, TRIM50, TRIM16, RFPL3, TRIM15, TRIM10, TRI	1095
TRIM16L, TRIM65, TRIM48, HNRNPUL2, TRIM43, TRIM50, TRIM16,	1095

Genes	List Tota
HPCAL1, TNNC1, NCALD, VSNL1, NCS1, KCNIP3	1095
HPCAL1, LPCAT1, NCALD, VSNL1, NCS1, MYL12A, KCNIP3	98
S100A6, HPCAL1, S100A8, TNNC1, NCALD, S100A9, S100A11, NCS1	1095

Genes	List Tota
LCN2, LCN15, ORM1, RBP1, LCN9, PAEP, ORM2	1095
LCN2, LCN15, ORM1, RBP1, LCN9, PAEP, ORM2	1095
LCN2, LCN15, ORM1, LCN9, PAEP, ORM2	1095

Genes	List Tota
MRPL11, MRPL1, MRPL51, RPL30, MRPL15, RPL7, MRPL16, RPL39L,	908
MRPL11, MRPL1, MRPL51, MRPL16, MRPL36	908
MRPL11, MRPL1, MRPL51, MRPL16, MRPL36	908

Genes	List Tota
PPIAL4C, PPIF, PPIH, PPIAL4G, PPIA	561
PPIAL4C, PPIF, PPIE, PPIH, PPIAL4G, PPIA	1206
PPIAL4C, PPIF, PPIE, PPIH, PPIAL4G, PPIA	1095

Genes	List Tota
NXN, PRDX6, PRDX4, PRDX1	1095

PRDX6, PRDX4, PRDX1, SOD3	1208
MGST3, LPO, PTGS2, NXN, PRDX6, PRDX4, PRDX1, SOD3	853
PRDX6, PRDX4, PRDX1	1095
PRDX6, PRDX4, PRDX1	561

Genes	List Tota
RPL35A, RRP1, NHP2L1, SNRPD3, LSM6, SNRPD1, NOP10, MRTO4, F	924
RPP38, PUS3, PUS1, LSM6, VARS, KARS, RPL7, NPM3, RPL5, MARS	924
RPL35A, RRP1, NHP2L1, LSM6, NOP10, MRTO4, RPS7, PA2G4, RPL7	924
RPL35A, PA2G4, RRP1, RPL7, LSM6, NPM3, RPL5, NOP56, PES1, T	924

Genes	List Tota
STEAP3, TFR2, TTYH1, MFI2, TRPC7, SLC39A7, SLC30A3, SLC39A4	924
TTYH1, SLC30A3, SLC39A4, TRPM2, SLC39A2, SLC39A1	853
SLC30A3, SLC39A4, SLC39A2, SLC39A1	853

Pop Hits	Pop Total	Fold Enri	Bonferron	Benjamini	FDR
64	19113	8.4193874	1.0564891	1.0564891	7.132188281824524E
64	19113	8.4193874	1.0564891	1.0564891	7.132188281824524E
70	19113	7.9241293	2.9048763	1.4524381	1.9610353478856754
70	19113	7.9241293	2.9048763	1.4524381	1.9610353478856754
70	19113	7.9241293	2.9048763	1.4524381	1.9610353478856754
71	19113	7.8125218	5.2314497	1.7438165	3.531667712729469E
73	19113	7.5984802	1.6341815	4.0854538	1.1032097244684905
78	19235	7.1449418	4.3351841	4.3351841	1.1154005755248876
75	19113	7.1845439	6.0112973	1.2022594	4.0581302635382673
72	16659	6.9729452	2.6877733	2.6877733	3.1241013989147577
72	16659	6.9729452	2.6877733	2.6877733	3.1241013989147577
61	7396	6.9159872	1.1112727	1.1112727	2.967166883109699E
73	16659	6.6690185	4.9838673	2.4919336	5.792938880072461E
28	16659	10.323581	4.4142467	1.4713785	5.129230373768223E
634	12983	2.3046660	1.0983214	1.0983214	1.872946242542639E
33	19113	9.1247550	2.4129098	4.0215164	1.6289192217300297
144	19235	3.6490238	1.4689625	2.9379251	3.7794974927507496
183	12782	2.8461881	7.4871999	2.4957395	2.406362427098685E
88	12782	3.8392070	1.2801902	2.5603935	4.1145016038690585
187	12782	2.7853070	1.3475460	2.2459227	4.330983063294269E
33	16659	5.5322540	0.0066585	8.3474932	0.0077650275190466

Pop Hits	Pop Total	Fold Enri	Bonferron	Benjamini	FDR
199	13528	4.4142791	1.4094234	1.4094234	9.118733031280933E
184	13528	4.4558629	2.7654219	1.3827109	1.7891815684033596
40	19235	9.1557326	6.3282712	2.1094237	1.6653345369377348
43	13528	8.1715493	9.1826546	3.0608848	5.995204332975845E
66	13528	6.4330316	9.1826546	2.2959412	5.995204332975845E
72	13528	6.1002886	1.8365309	3.6726177	1.1879386363489175
25	16659	10.345315	1.7783108	4.4457770	2.0670132272471164
137	13528	3.7403229	6.6159493	1.1026582	4.280408250068035E
227	13528	2.5798577	2.0179439	2.2423610	1.3057067272947975
22	12782	7.0385462	3.9920361	4.4363828	0.0012832710851506

Pop Hits	Pop Total	Fold Enri	Bonferron	Benjamini	FDR
279	19235	3.4243039	6.3282712	3.1641356	1.6653345369377348
515	12782	2.2960694	3.5660363	3.5660363	1.1461054327810416
188	19235	3.3031782	6.2474653	1.5618663	1.6074118702746887
168	12983	3.2615139	6.7130685	3.3565348	1.1449579728761705
215	12782	2.6844688	6.3625535	3.1812818	2.044903516518204E
48	12782	4.9856369	4.1455379	5.9223023	1.3323847221080598
48	12782	4.9856369	4.1455379	5.9223023	1.3323847221080598

331	13528	2.0788898	0.0080812	4.5067803	0.0052495054540413
128	12782	2.8594094	0.0013022	1.3030375	0.0041880916989756
63	12782	3.1282427	0.1610234	0.0109132	0.5626932482249769
18	12782	5.4744248	0.3867037	0.0241489	1.5590458791401973
18	12782	5.4744248	0.3867037	0.0241489	1.5590458791401973

Pop Hits	Pop Total	Fold Enri	Bonferron	Benjamini	FDR
515	12782	2.2960694	3.5660363	3.5660363	1.146105432781041
547	13528	1.6594569	0.2235317	0.0114341	0.1635525869393084
718	12983	1.4838828	0.5242262	0.0600241	1.258924164431996

Pop Hits	Pop Total	Fold Enri	Bonferron	Benjamini	FDR
47	5085	6.6579378	2.5325759	2.5325759	1.872529908908404
56	19235	5.6867904	3.2369083	4.6241553	8.328250533651271E
61	12782	4.6154401	9.2161509	2.3040457	2.962044319820833
65	13528	4.5048285	9.8251955	1.4036584	6.357046776761521E
65	13528	4.5048285	9.8251955	1.4036584	6.357046776761521E
67	13528	4.3703560	1.6900602	2.1127314	1.093532388973805
67	13528	4.3703560	1.6900602	2.1127314	1.093532388973805
19	16659	8.8079307	1.0883401	1.5548441	1.265087283597310
68	13528	4.3060860	2.1971484	2.1973657	1.421673331614137
20	19235	8.7576572	5.3812873	6.7267675	1.384589016550919E
70	13528	4.1830550	3.6522449	3.3207740	2.363369972280260
20	12983	8.3712192	1.3002960	4.3345079	2.217881362787110
20	12983	8.3712192	1.3002960	4.3345079	2.217881362787110
71	13528	4.1241387	4.6715672	3.8938064	3.023127152945726E
17	19235	9.3664783	1.2819903	1.4245148	3.298642072491198E
73	13528	4.0111486	7.5290587	5.7935972	4.872997413696289
20	12782	7.7424008	1.3006637	1.6259221	4.180540143949862
74	13528	3.9569439	9.4896853	6.7813349	6.142563211297691E
84	13528	3.6601731	0.0017546	1.1707213	0.0011362156030703
78	13528	3.7540237	0.0022901	1.4328936	0.0014833882629794
81	13528	3.6149858	0.0042453	2.5022944	0.002752508796177
100	13528	3.0745454	0.0304271	0.0016249	0.0199895904023250
14	16659	8.6935420	0.0165843	0.0013926	0.019436371631254
102	13528	3.0142602	0.0412051	0.0021016	0.0272201311807496
102	13528	3.0142602	0.0412051	0.0021016	0.0272201311807496
12	16659	8.8746575	0.0709609	0.0045897	0.0855167179338423
370	13528	1.8201942	0.2590433	0.0129507	0.1937860519162115
119	13528	2.5836516	0.3376822	0.0170205	0.2662085381515799
19	19235	5.8663733	0.3680589	0.0181908	1.1739123725532319
8	16659	9.5085616	0.7735279	0.0600048	1.7114138231755005
187	13528	1.9573118	0.9964795	0.1769996	3.5889269895025055
565	13528	1.4770256	0.9997230	0.2086787	5.161954527014611
187	13528	1.8790193	0.9999931	0.2628109	7.405105354798113
180	13528	1.8707551	0.9999997	0.3006622	9.470217644753786

776	13528	1.3584147	0.9999999	0.329675	10.99268269679714
334	13528	1.5780387	0.9999999	0.3436152	12.49451829394359
277	13528	1.5856345	1.0	0.4725574	22.312037173222976
242	13528	1.5729669	1.0	0.6188675	36.195179218660165
734	13528	1.2566262	1.0	0.7726653	56.560688977961405
233	13528	1.5080541	1.0	0.7773417	57.89239431192434
295	13528	1.4392545	1.0	0.7749229	58.036441322283515

Pop Hits	Pop Total	Fold Enri	Bonferron	Benjamini	FDR
27	16659	7.8885844	4.7193008	9.4386196	5.485436005425726E
28	16659	7.6068493	8.2802848	1.3800522	9.624529861973485E
21	19113	9.0561478	3.0821810	4.4031739	2.0807627842955867
19	7396	8.3264846	1.0049861	5.0249433	2.6833882160737232
18	19113	8.8045881	0.0011608	1.4518062	7.841266959407456E
60	19235	3.4499862	0.1561180	0.0076858	0.4357788753608149
182	19113	2.0028019	0.9987274	0.3407611	4.4008139766111505
182	19113	1.9157235	0.9999993	0.5091911	9.161913419398537
236	16659	1.6116206	0.9999999	0.5045342	30.740998468395876
130	16659	1.8724552	0.9999999	0.5194836	32.42016447430917
215	16659	1.6275119	0.9999999	0.5312858	35.05180197751066
217	16659	1.6125118	1.0	0.5501545	37.72144146870418

Pop Hits	Pop Total	Fold Enri	Bonferron	Benjamini	FDR
15	1437	10.191489	6.8756174	6.8756174	5.829042953875429
126	5085	3.1043956	1.4300931	7.1507214	0.0010574490856951
17	9079	9.3512245	3.5424579	3.5424579	0.0019535348669830
209	19235	2.5141599	0.0014332	1.3038285	0.0036902585672904
17	16659	8.0543110	0.0067982	7.5765862	0.0079285891066649
19	16659	7.2064888	0.0186611	0.0014479	0.0218930671468386
24	12782	5.8654552	0.0094843	7.9382265	0.0306232089245739
260	19235	2.1434825	0.0222044	0.0014024	0.0577570159406848
284	13528	2.0105176	0.1273205	0.0064641	0.0880718903715327
547	13528	1.6594569	0.2235317	0.0114341	0.1635525869393084
120	19235	2.6538355	0.0916979	0.0045694	0.2471515231284193
321	13528	1.8243853	0.5787398	0.0339891	0.557758733613456
132	12782	2.3461820	0.1561997	0.0112587	0.5443695808756255
153	13528	2.2008884	0.8531137	0.0711174	1.2333080295425902
153	13528	2.2008884	0.8531137	0.0711174	1.2333080295425902
153	13528	2.2008884	0.8531137	0.0711174	1.2333080295425902
370	13528	1.6223470	0.9991072	0.1916522	4.4409527212318505
540	19235	1.5038401	0.8871703	0.0659110	5.459090919896148
28	13528	3.6601731	0.9999999	0.4023501	16.737265714412086

Pop Hits	Pop Total	Fold Enri	Bonferron	Benjamini	FDR
22	16659	6.9153175	0.0070864	7.1091500	0.0082658328457441

16	9079	8.8317120	0.0030050	0.0010027	0.0165927581457836
20	19113	7.1317164	0.0399424	0.0045188	0.0275139916879552
16	16659	7.6068493	0.0483915	0.0033013	0.0576371829937194
18	16659	6.7616438	0.1155665	0.0067994	0.1426425684272869
8	7396	8.2397504	0.6217480	0.2767967	2.56241831635724
126	19235	2.1483430	0.9569982	0.0815253	7.776642797587541

Pop Hits	Pop Total	Fold Enri	Bonferron	Benjamini	FDR
13	16659	9.3622760	0.0088260	8.0560310	0.0103038352688433
92	12983	3.3087823	0.0059414	0.0014886	0.0101632028089992
15	9079	9.4204928	0.0017776	8.8921434	0.0098096177281736
153	12983	2.5864729	0.0180372	0.0036337	0.0310397703505826
15	16659	8.1139726	0.0291441	0.0021104	0.0343729223170052
80	19235	3.3836403	0.0175528	0.0012641	0.0455525158395642
145	12983	2.5192383	0.0603422	0.0103196	0.1060973565218614
107	19235	2.8274509	0.0622184	0.0033752	0.1651421491924876
8	16659	9.5085616	0.7735279	0.0600048	1.7114138231755005
45	19113	3.5218352	0.9891761	0.2762331	3.0092258162210284
10	19113	7.9241293	0.9983169	0.3467599	4.220229552961463
270	12983	1.7475272	0.9473845	0.1509158	4.89841438077635
40	9079	3.5326848	0.7793564	0.2226524	7.994655473414025
33	7396	3.1960244	0.9966396	0.6129814	14.107922505142835
40	16659	3.0427397	0.9999999	0.4248351	21.16935748088301
5	19113	9.5089552	1.0	0.9461488	46.8009336623934
5	19113	9.5089552	1.0	0.9461488	46.8009336623934

Pop Hits	Pop Total	Fold Enri	Bonferron	Benjamini	FDR
116	9079	3.0454179	0.0055651	0.0013942	0.0307656107350728
136	19235	2.6928625	0.0215674	0.0014525	0.0560823819511391
106	19113	2.8407256	0.2422270	0.0273560	0.1870738110201886
104	16659	2.7794257	0.1756105	0.0101123	0.2242099645241801
105	16659	2.7529549	0.1966931	0.0108911	0.2542498291643214
154	12983	2.3720101	0.1467357	0.0224144	0.2702834436653978
116	16659	2.6230514	0.2368069	0.0127863	0.3136224377767949
178	12983	2.1376964	0.4223856	0.0591609	0.9317297826890392
196	19113	2.1023200	0.8023133	0.1263621	1.0883935152434798
180	12983	2.1139442	0.4756426	0.0570002	1.0950422646154645
375	12983	1.7046846	0.5498580	0.0595524	1.3521444153651418
574	12983	1.4053678	0.9999498	0.375878	915.535987646977157
484	19235	1.4475466	0.9996694	0.166528	718.634159959722652
549	12983	1.3307452	0.9999999	0.6672925	43.05521292394378

Pop Hits	Pop Total	Fold Enri	Bonferron	Benjamini	FDR
1689	19235	1.4424043	0.0015399	1.2841975	0.0039651138010632
3250	19235	1.1121612	0.9999999	0.3685206	48.43531324779773

2010	12782	1.1345716	0.9999999	0.3899015	49.48476342343907

Pop Hits	Pop Total	Fold Enri	Bonferron	Benjamini	FDR
38	19235	4.1902666	0.2285041	0.0112159	0.6652500228503277
32	19113	3.9620646	0.9998083	0.3955999	5.614808074462118
35	16659	3.4774168	0.9999429	0.2498013	10.737243481991342

Pop Hits	Pop Total	Fold Enri	Bonferron	Benjamini	FDR
1779	12782	1.2898066	0.1384611	0.0105889	0.4778470150879288
1856	12782	1.2742195	0.1843194	0.0119127	0.6526483473191846
1820	12782	1.2762199	0.1964538	0.0120776	0.7004936322468125
698	12782	1.2705685	0.9999999	0.3574640	42.56054410693797
1450	12782	1.1650007	0.9999999	0.3897703	48.66121596624928

Pop Hits	Pop Total	Fold Enri	Bonferron	Benjamini	FDR
36	16659	4.6486301	0.1016046	0.0062828	0.1244612010958046
10	16659	9.1282191	0.2794869	0.0147891	0.3802799187404115
8	19235	9.9518832	0.3946857	0.0191227	1.2833108019152162
42	19235	3.7911936	0.4322661	0.0207484	1.445970426631904
26	16659	4.6811380	0.7976032	0.0619021	1.8397311322268406
9	19113	8.8045881	0.9822437	0.2666103	2.6845805397576084
9	19113	8.8045881	0.9822437	0.2666103	2.6845805397576084
9	12983	8.4557769	0.8039438	0.1098652	2.7407117443533524
9	12983	8.4557769	0.8039438	0.1098652	2.7407117443533524
38	12983	3.6048312	0.9225758	0.1477740	4.269778944902713
10	12983	7.6101992	0.9239683	0.1406380	4.299406601868094
63	13528	2.7887033	0.9998812	0.2220344	5.680139189177846
6	16659	10.142465	0.9990099	0.2059364	7.725992908856039
7	19113	9.0561478	0.9999999	0.5791709	12.062773483756173
7	7396	7.5334861	0.9986255	0.5612011	16.133960441916773
101	16659	2.1088295	0.9999999	0.4271074	21.811351788447418
4	19113	11.886194	1.0	0.8745871	32.4593130663313
4	19113	11.886194	1.0	0.8745871	32.4593130663313
4	16659	11.410273	0.9999999	0.5147476	32.633422127261454
80	12782	2.1115638	0.9999868	0.2815331	30.32304511536774
27	16659	3.3808219	1.0	0.5543132	38.642446859688704
12	19235	5.3076710	0.9999999	0.3212318	41.04387140347407

Pop Hits	Pop Total	Fold Enri	Bonferron	Benjamini	FDR
21	19235	5.3076710	0.5613514	0.0270947	2.0978990959901633
35	13528	3.7647495	0.9964992	0.1717931	3.5924255819685835
97	13528	2.4149596	0.9982207	0.1847371	4.013659137496006
31	5085	4.0377171	0.3660526	0.0871264	3.313852654311389
23	13528	4.4558629	0.9999597	0.2392809	6.337226105884741

23	13528	4.4558629	0.9999597	0.2392809	6.337226105884741
31	13528	3.7782432	0.9999898	0.2610531	7.1679203961284355
31	13528	3.7782432	0.9999898	0.2610531	7.1679203961284355
26	13528	3.9417249	0.9999999	0.3325172	11.7977790933246
144	13528	1.9317580	0.9999999	0.3747944	15.133496591520823
4	12983	11.415298	0.9999999	0.5833666	31.155947008073248

Pop Hits	Pop Total	Fold Enri	Bonferron	Benjamini	FDR
832	19235	1.7033030	5.2991139	5.3003780	0.0013637623799356
595	12782	1.6324695	0.0248283	0.0019321	0.0807718441810267
476	19235	1.7394888	0.0726079	0.0037618	0.1937545502993520
128	5085	2.4447115	0.0655150	0.0223334	0.4997479321285913
467	19113	1.6628793	0.7857589	0.1306934	1.0346805318848906
227	12782	1.9224223	0.2654975	0.0161089	0.986803404262504
227	12782	1.9224223	0.2654975	0.0161089	0.986803404262504
97	13528	2.4149596	0.9982207	0.1847371	4.013659137496006
1087	12782	1.3209415	0.6902918	0.0518839	3.697084084079283
620	12782	1.4304142	0.8005659	0.0676982	5.049807184316501
622	12782	1.4258148	0.8240726	0.0698445	5.431752618908126
193	19235	1.8975611	0.9391913	0.0790537	6.950806811512111
394	12782	1.5363324	0.9029399	0.0890770	7.222230658734108
419	12782	1.5118595	0.9173703	0.0914446	7.700954647323632
125	12782	2.0271013	0.9563686	0.1095240	9.575997865393926
144	13528	1.9317580	0.9999999	0.3747944	15.133496591520823
100	19235	2.2292218	0.9959994	0.128929	13.242909854553153
180	5085	1.8253846	0.8057267	0.2389692	11.409562203436252
329	12782	1.5403505	0.9906725	0.1488780	13.950367427380606
64	13528	2.5163690	0.9999999	0.417226	17.768543915756275
64	12782	2.4195002	0.9980552	0.1878637	18.178889809116782
75	12782	2.2523348	0.9992507	0.2013941	20.649028104697244
306	12782	1.4721142	0.9999972	0.2990966	33.713861166654524
71	19235	2.2426779	0.9999999	0.3071297	38.79221139416488
56	13528	2.3529684	1.0	0.7132640	47.19074701695681
56	13528	2.3529684	1.0	0.7132640	47.19074701695681
130	5085	1.8053254	0.9975614	0.4875139	35.90696197099196
313	13528	1.4500366	1.0	0.7279907	49.02607246895899
49	12983	2.4849630	0.9999999	0.6734699	46.737909985824345
114	13528	1.7979797	1.0	0.7764283	57.38195324819595

Pop Hits	Pop Total	Fold Enri	Bonferron	Benjamini	FDR
24	16659	5.0712328	0.6106404	0.0401813	1.0903894329109765
22	16659	4.8407222	0.9656207	0.1215770	3.8416924442175038
23	16659	4.6302561	0.9865822	0.1475777	4.887563464185285
24	16659	4.4373287	0.9956413	0.1764478	6.122552383406132
22	7396	4.1947820	0.9274771	0.4810581	6.766073630719616
49	13528	2.9878964	0.9999996	0.2959841	9.09597339129009

9	16659	6.7616438	0.9999999	0.4590869	25.3861229447771
162	13528	1.7171182	1.0	0.6490770	39.42936252193522

Pop Hits	Pop Total	Fold Enri	Bonferron	Benjamini	FDR
137	12983	2.3330537	0.4260778	0.0540127	0.9425643988136501
111	19235	2.4386596	0.5778386	0.0274349	2.194353785034231
52	12983	3.2196997	0.8050664	0.1032753	2.7502364909707255
95	12983	2.4032208	0.9594084	0.1551886	5.3183197239070275
24	12983	3.8050996	0.9999999	0.4996376	24.680663903708332
20	12983	3.8050996	0.9999999	0.6740974	45.76837418381092
20	16659	3.8034246	1.0	0.6306942	47.71133674353633

Pop Hits	Pop Total	Fold Enri	Bonferron	Benjamini	FDR
10	12983	7.6101992	0.9239683	0.1406380	4.299406601868094
11	19113	7.2037539	0.9999284	0.4115511	6.24031789053473
11	19113	7.2037539	0.9999284	0.4115511	6.24031789053473
11	16659	6.9153175	0.9974688	0.1863093	6.713715197839187
12	19235	6.6345888	0.9452190	0.0796334	7.200386939347014
19	19235	5.0283199	0.9530250	0.0814402	7.566708161112734
12	16659	6.3390410	0.9997994	0.2401660	9.422571656974899
12	16659	6.3390410	0.9997994	0.2401660	9.422571656974899
11	7396	5.9925457	0.9781648	0.5345947	9.706924810907115
57	19235	2.7935110	0.9928570	0.1190094	11.93924487373973
8	19235	7.9615066	0.9980320	0.1409854	14.812124781671166
8	19235	7.9615066	0.9980320	0.1409854	14.812124781671166
10	19113	6.3393034	1.0	0.8790076	31.95254870120091
10	19113	6.3393034	1.0	0.8790076	31.95254870120091

Pop Hits	Pop Total	Fold Enri	Bonferron	Benjamini	FDR
19	9079	5.5779234	0.5465689	0.1463059	4.267140377207612
19	16659	4.8043258	0.9999024	0.2506952	10.17867510610998
19	16659	4.8043258	0.9999024	0.2506952	10.17867510610998
19	16659	4.8043258	0.9999024	0.2506952	10.17867510610998
19	7396	4.1632423	0.9983370	0.5991484	15.706145900017265
21	13528	4.1830550	0.9999999	0.4333904	19.19844799279672

Pop Hits	Pop Total	Fold Enri	Bonferron	Benjamini	FDR
42	13528	3.1372912	0.9999999	0.3272164	11.353830427203338
42	13528	3.1372912	0.9999999	0.3272164	11.353830427203338
42	13528	3.1372912	0.9999999	0.3272164	11.353830427203338
43	13528	3.0643310	0.9999999	0.3502930	13.020745988555948
43	13528	3.0643310	0.9999999	0.3502930	13.020745988555948

Pop Hits	Pop Total	Fold Enri	Bonferron	Benjamini	FDR
25	5085	5.0067692	0.1094301	0.0285577	0.8532316613421242
109	13528	2.4177290	0.9516186	0.1061094	1.940407325415483
58	13528	3.0291088	0.9895115	0.1502111	2.9055688096447763
79	13528	2.5945531	0.9990159	0.1945616	4.380727085395952
71	13528	2.6806902	0.9996493	0.2086301	5.016950944962472
85	13528	2.4114081	0.9999983	0.2831162	8.253163489029802
40	13528	3.2941558	0.9999988	0.2836680	8.469216559992654
40	13528	3.2941558	0.9999988	0.2836680	8.469216559992654
41	13528	3.2138105	0.9999998	0.3049693	9.838140120427353
69	13528	2.5462074	0.9999999	0.3310000	11.275277148234853
42	13528	3.1372912	0.9999999	0.3272164	11.353830427203338
153	13528	1.9138160	0.9999999	0.3553328	13.485662707981238
81	13528	2.3497407	0.9999999	0.3652433	14.179524506029706
3	19235	15.923013	0.9984719	0.1430496	15.36487883192298
9	13528	6.5069745	1.0	0.5772872	29.599412377263036
16	13528	4.5752164	1.0	0.5854018	30.550163467826497
192	13528	1.6775793	1.0	0.6049452	32.33273826437121
52	13528	2.5339660	1.0	0.6150913	34.702579277030864
11	13528	5.3238882	1.0	0.7114698	46.5943586866337
56	13528	2.3529684	1.0	0.7132640	47.19074701695681
47	13528	2.4920327	1.0	0.7356609	50.632467029135974
222	13528	1.5168285	1.0	0.7832842	59.755573466592594

Pop Hits	Pop Total	Fold Enri	Bonferron	Benjamini	FDR
6	19113	10.565505	0.9999887	0.4511156	7.4056980838541175
6	19113	10.565505	0.9999887	0.4511156	7.4056980838541175
5	7396	7.9101604	0.9999999	0.9359107	51.981974459884796

Pop Hits	Pop Total	Fold Enri	Bonferron	Benjamini	FDR
6	1437	10.191489	0.4696535	0.2717511	5.234847994548131
12	1437	6.3696808	0.5319486	0.2235779	6.233419428538845
12	1437	5.0957446	0.9951922	0.6561349	36.39678343414137

Pop Hits	Pop Total	Fold Enri	Bonferron	Benjamini	FDR
26	12983	4.0977996	0.9951848	0.2341737	8.699043907444015
14	13528	5.2288188	0.9999999	0.4419694	19.962363359096468
12	12983	5.0734661	0.9999999	0.6638892	46.86130388103569

Pop Hits	Pop Total	Fold Enri	Bonferron	Benjamini	FDR
70	19235	2.9571310	0.5152847	0.0246629	1.846028113102871
90	12782	2.0333578	0.9999844	0.2850605	29.94531792546844
104	12782	1.8949932	0.9999994	0.3226012	37.07166958647179
108	12782	1.8248082	0.9999999	0.3687498	45.45925421584089

Pop Hits	Pop Total	Fold Enri	Bonferron	Benjamini	FDR
41	9079	3.4465217	0.8239052	0.1951437	9.131647174806501
41	16659	2.9685265	0.9999999	0.4503813	23.76282893385383
48	16659	2.5356164	1.0	0.6186927	45.401330218025315

Pop Hits	Pop Total	Fold Enri	Bonferron	Benjamini	FDR
62	16659	2.6992045	0.9999355	0.2535456	10.61026172668289
72	9079	2.6985786	0.8010561	0.2060033	8.518336723152508
55	19113	2.8815015	0.9999999	0.5862866	11.761034355078227
210	19113	1.7357616	0.9999999	0.7720047	21.300669021951357
82	19113	2.3192573	0.9999999	0.7643310	21.645696534036286
48	9079	2.9439040	0.9829909	0.3346224	20.118735999609918
72	16659	2.3243150	0.9999999	0.4729308	26.848916038565164
87	16659	2.0984411	1.0	0.5373696	36.108304657957014
17	7396	3.8775296	0.9999999	0.8850835	40.5427814463634
48	16659	2.5356164	1.0	0.6186927	45.401330218025315
84	16659	1.9922700	1.0	0.7006095	55.649079310084424

Pop Hits	Pop Total	Fold Enri	Bonferron	Benjamini	FDR
22	16659	4.1491905	0.9999999	0.3903066	18.70166175589797
43	1950	3.2392026	0.3008021	0.3008021	12.730360058401558
130	16659	1.8724552	0.9999999	0.5194836	32.42016447430917

Pop Hits	Pop Total	Fold Enri	Bonferron	Benjamini	FDR
32	16659	3.3279965	0.9999999	0.4449050	23.941572855752447
33	16659	3.2271481	0.9999999	0.4694874	27.154721859104725
25	16659	3.6512876	0.9999999	0.5020932	29.997625967836584

Pop Hits	Pop Total	Fold Enri	Bonferron	Benjamini	FDR
67	12782	2.5212703	0.9566907	0.1060639	9.597533062227603
18	12782	3.9103034	0.9999998	0.3349895	39.23972307928963
18	12782	3.9103034	0.9999998	0.3349895	39.23972307928963

Pop Hits	Pop Total	Fold Enri	Bonferron	Benjamini	FDR
7	7396	7.5334861	0.9986255	0.5612011	16.133960441916773
18	19113	4.4022940	1.0	0.8854507	34.5703200867837
21	16659	3.6223091	1.0	0.6840275	53.387621275348216

Pop Hits	Pop Total	Fold Enri	Bonferron	Benjamini	FDR
7	16659	8.6935420	0.9999902	0.2807927	12.548831762072531

9	19235	7.0768947	0.9998683	0.1800984	20.539231531199498
47	12983	2.5907061	0.9999999	0.6461841	40.18378116939192
5	16659	9.1282191	1.0	0.6293470	46.979116583938875
5	7396	7.9101604	0.9999999	0.9359107	51.981974459884796

Pop Hits	Pop Total	Fold Enri	Bonferron	Benjamini	FDR
180	13528	1.7894179	0.9999999	0.4174751	18.068187878528363
230	13528	1.5913796	1.0	0.6225945	36.0849079804306
122	13528	1.8000851	1.0	0.7350679	50.1471524173341
92	13528	1.9096555	1.0	0.7759328	58.54927906090887

Pop Hits	Pop Total	Fold Enri	Bonferron	Benjamini	FDR
71	13528	2.2682763	1.0	0.6001455	32.39037085425197
27	12983	3.3823107	0.9999999	0.6321775	36.907860345151086
12	12983	5.0734661	0.9999999	0.6638892	46.86130388103569

Log P-value
-18.9761
-18.9761
-18.8379
-18.8379
-18.8379
-18.7585
-18.3888
-17.363
-16.92
-15.5706
-15.5706
-15.9542
-14.6035
-11.8323
-10.9593
-10.3956
-7.53196
-5.6028
-5.59169
-5.64861
-3.07844
-18.851
-17.8593
-13.6758
-12.5142
-12.639
-12.435
-10.3521
-7.95756
-4.64929
-4.35297
-13.4997
-9.44781
-7.80636
-6.47411
-5.4974
-5.22751
-5.22751

-3.34613
-3.88504
-1.96204
-1.6171
-1.6171

-9.44781
-1.9418
-1.22167

-8.59644
-7.33497
-5.63751
-4.85274
-4.85274
-4.67516
-4.67516
-4.80831
-4.6581
-5.17219
-4.47876
-4.36306
-4.36306
-4.40963
-4.84633
-4.23705
-4.7889
-4.16868
-3.93155
-3.84379
-3.60166
-2.78915
-2.85616
-2.67743
-2.67743
-2.33821
-1.8877
-1.76903
-1.74015
-1.22181
-0.75203
-0.68052
-0.58036
-0.52192

-0.48192
-0.46393
-0.32555
-0.2084
-0.11201
-0.10939
-0.11074

-6.02509
-5.8601
-5.35623
-5.29887
-3.83809
-2.11431
-0.46755
-0.29312
-0.29711
-0.28443
-0.27467
-0.25952

-5.16269
-4.14565
-3.4507
-3.88478
-3.12053
-2.83924
-3.10028
-2.85312
-2.18949
-1.9418
-2.34014
-1.46866
-1.94851
-1.14802
-1.14802
-1.14802
-0.71749
-1.18104
-0.3954

-3.14818

-2.99883
-2.34497
-2.48131
-2.16753
-0.55784
-1.08871

-3.09388
-2.8272
-3.05099
-2.43964
-2.67563
-2.89821
-1.98634
-2.47169
-1.22181
-0.55872
-0.45997
-0.82127
-0.65237
-0.21255
-0.37178
-0.02404
-0.02404

-2.85567
-2.83788
-1.56295
-1.99515
-1.96292
-1.64947
-1.89325
-1.22796
-0.89838
-1.24412
-1.2251
-0.42495
-0.77851
-0.17568

-3.89137
-0.43354

-0.40905

-1.95016
-0.40274
-0.60241

-1.97515
-1.92399
-1.91802
-0.44677
-0.40919

-2.20184
-1.83006
-1.71845
-1.68301
-1.20829
-0.57412
-0.57412
-0.95914
-0.95914
-0.8304
-0.8519
-0.65358
-0.68627
-0.23719
-0.25088
-0.36946
-0.0582
-0.0582
-0.28841
-0.55047
-0.25624
-0.49318

-1.56712
-0.76499
-0.73345
-1.05985
-0.62109

-0.62109
-0.58327
-0.58327
-0.47819
-0.42621
-0.23406

-4.27569
-2.71397
-2.4246
-1.65104
-0.88375
-1.79293
-1.79293
-0.73345
-1.28497
-1.16942
-1.15587
-1.10208
-1.05023
-1.03884
-0.96049
-0.42621
-0.88965
-0.62166
-0.82717
-0.37963
-0.72616
-0.69595
-0.52419
-0.51268
-0.14675
-0.14675
-0.31201
-0.13787
-0.17168
-0.1099

-1.39598
-0.91515
-0.83098
-0.75338
-0.3178
-0.52873

-0.33811
-0.1877

-1.2675
-1.5617
-0.986
-0.80914
-0.30134
-0.17128
-0.20018

-0.8519
-0.38558
-0.38558
-0.72977
-1.0989
-1.08916
-0.61949
-0.61949
-0.27198
-0.92442
-0.85083
-0.85083
-0.05601
-0.05601

-0.83474
-0.60085
-0.60085
-0.60085
-0.22247
-0.36312

-0.48516
-0.48516
-0.48516
-0.45557
-0.45557

-1.54428
-0.97425
-0.8233
-0.71094
-0.68062
-0.54804
-0.54719
-0.54719
-0.51574
-0.48017
-0.48516
-0.44936
-0.43742
-0.84451
-0.23861
-0.23255
-0.21828
-0.21106
-0.14784
-0.14675
-0.13332
-0.10608

-0.34571
-0.34571
-0.02877

-0.56583
-0.65057
-0.18301

-0.63046
-0.35461
-0.1779

-1.60795
-0.54506
-0.49133
-0.43327

-0.70965
-0.34642
-0.20853

-0.59594
-0.68613
-0.23189
-0.11238
-0.11672
-0.47544
-0.3252
-0.26973
-0.05302
-0.20853
-0.15452

-0.40859
-0.52172
-0.28443

-0.35173
-0.32838
-0.29922

-0.97443
-0.47497
-0.47497

-0.25088
-0.05284
-0.16493

-0.55161

-0.74449
-0.18964
-0.20111
-0.02877

-0.37937
-0.20579
-0.13367
-0.11018

-0.22174
-0.19916
-0.1779
